# An International, Multidisciplinary Consensus Set of Patient-Centered Outcome Measures for Substance-Related and Addictive Disorders

**DOI:** 10.3390/jcm13072154

**Published:** 2024-04-08

**Authors:** Nicola Black, Sophie Chung, Calvert Tisdale, Luz Sousa Fialho, Apinun Aramrattana, Sawitri Assanangkornchai, Alex Blaszczynski, Henrietta Bowden-Jones, Wim van den Brink, Adrian Brown, Qiana L. Brown, Linda B. Cottler, Maury Elsasser, Marica Ferri, Maria Florence, Ralitza Gueorguieva, Ryan Hampton, Suzie Hudson, Peter J. Kelly, Nicholas Lintzeris, Lynette Murphy, Abhijit Nadkarni, Joanne Neale, Daniel Rosen, Hans-Jürgen Rumpf, Brian Rush, Gabriel Segal, Gillian W. Shorter, Marta Torrens, Christopher Wait, Katherine Young, Michael Farrell

**Affiliations:** 1 National Drug and Alcohol Research Centre, UNSW, Sydney 2052, Australia; 2 International Consortium for Health Outcomes Measurement, London W12 8EU, UK; 3 Department of Family Medicine, Faculty of Medicine, Chiang Mai University, Chiang Mai 50200, Thailand; apinun.aramrat@gmail.com; 4 Department of Epidemiology, Faculty of Medicine, Prince of Songkla University, Songkhla 90110, Thailand; 5 School of Psychology, Brain and Mind Centre, Faculty of Science, University of Sydney, Sydney 2006, Australia; alex.blaszczynski@sydney.edu.au; 6 Department of Psychiatry, University College London, London NW1 2AE, UK; 7 Department of Psychiatry, Cambridge University, Cambridge CB2 1QW, UK; 8 Amsterdam University Medical Centers, Department of Psychiatry, University of Amsterdam, 1105 AZ Amsterdam, The Netherlands; 9 Northwick Park Hospital, Central and North West London Trust, London HA1 3UJ, UK; 10 School of Social Work, Rutgers, The State University of New Jersey, New Brunswick, NJ 08901, USA; 11 Department of Epidemiology, College of Medicine & Public Health and Health Professions, University of Florida, Homestead, FL 33031, USA; 12 Independent Researcher, South Berwick, ME 03908, USA; 13 Department of Psychology, Faculty of Community and Health, European Monitoring Centre for Drugs and Drug Addiction, 1249-289 Lisbon, Portugal; marica.ferri@emcdda.europa.eu; 14 Department of Biostatistics, School of Public Health, University of the Western Cape, Cape Town 7535, South Africa; mflorence@uwc.ac.za; 15 Department of Biostatistics, School of Public Health, Yale University, New Haven, CT 06511, USA; 16 The Voices Project, Las Vegas, NV 89149, USA; 17 Network of Alcohol and Other Drugs Agencies, Sydney 2751, Australia; suzie.hudson@health.nsw.gov.au; 18 Illawarra Health and Medical Research Institute, University of Wollongong, Wollongong 2522, Australia; pkelly@uow.edu.au; 19 Discipline of Addiction Medicine, Faculty of Medicine and Health, University of Sydney, Sydney 2006, Australia; nicholas.lintzeris@health.nsw.gov.au; 20 Independent Researcher, Sydney 2000, Australia; 21 Addictions and Related Research Group, Sangath, Bardez 403501, Goa, India; 22 Centre for Global Mental Health, Department of Population Health, London School of Hygiene and Tropical Medicine, London WC1E 7HT, UK; 23 Addictions Department, King’s College London, London SE1 9NH, UK; 24 School of Social Work, University of Pittsburgh, Pittsburgh, PA 15213, USA; 25 Translational Psychiatry Unit, Universität zu Lübeck, 23562 Lübeck, Germany; 26 Institute for Mental Health Policy Research, Centre for Addiction and Mental Health, Toronto, ON M6J 1H4, Canada; 27 Department of Philosophy, King’s College London, London SE1 9NH, UK; gabrielsegal@virginmedia.com; 28 Drug and Alcohol Research Network, School of Psychology, Queen’s University Belfast, Belfast BT9 5AJ, UK; 29 Institute of Mental Health Sciences, Ulster University, Coleraine BT52 1SA, UK; 30 Addiction Service, Hospital del Mar, 08003 Barcelona, Spain; 31 Build on Belief, London SW5 9HB, UK

**Keywords:** consensus set, outcome measures, patient-centered, Delphi, substance use, addictive disorders, addiction, gaming disorder, gambling disorder, ICHOM, core outcome set

## Abstract

**Background:** In 1990, the United States’ Institute of Medicine promoted the principles of outcomes monitoring in the alcohol and other drugs treatment field to improve the evidence synthesis and quality of research. While various national outcome measures have been developed and employed, no global consensus on standard measurement has been agreed for addiction. It is thus timely to build an international consensus. Convened by the International Consortium for Health Outcomes Measurement (ICHOM), an international, multi-disciplinary working group reviewed the existing literature and reached consensus for a globally applicable minimum set of outcome measures for people who seek treatment for addiction. **Methods:** To this end, 26 addiction experts from 11 countries and 5 continents, including people with lived experience (*n* = 5; 19%), convened over 16 months (December 2018–March 2020) to develop recommendations for a minimum set of outcome measures. A structured, consensus-building, modified Delphi process was employed. Evidence-based proposals for the minimum set of measures were generated and discussed across eight videoconferences and in a subsequent structured online consultation. The resulting set was reviewed by 123 professionals and 34 people with lived experience internationally. **Results:** The final consensus-based recommendation includes alcohol, substance, and tobacco use disorders, as well as gambling and gaming disorders in people aged 12 years and older. Recommended outcome domains are frequency and quantity of addictive disorders, symptom burden, health-related quality of life, global functioning, psychosocial functioning, and overall physical and mental health and wellbeing. Standard case-mix (moderator) variables and measurement time points are also recommended. **Conclusions:** Use of consistent and meaningful outcome measurement facilitates carer–patient relations, shared decision-making, service improvement, benchmarking, and evidence synthesis for the evaluation of addiction treatment services and the dissemination of best practices. The consensus set of recommended outcomes is freely available for adoption in healthcare settings globally.

## 1. Introduction

Tobacco smoking, alcohol use, and other substance use each represent one of the ten leading risk factors contributing to global disability-adjusted life-years worldwide [1,2]. Tobacco and alcohol use prevalence is consistently high globally, with over a billion tobacco smokers and high levels of alcohol consumption in most populations [2,3]. Furthermore, 3 million people die prematurely every year from the harmful use of alcohol, representing 5.3% of all deaths worldwide [4]. The UNODC World Drug Report 2023 estimates that in 2021; 1 in every 17 people aged 15–64 in the world had used a drug in the past 12 months, with the estimated number of illicit drug users growing from 240 million in 2011 to 296 million in 2021 (5.8% of the global population aged 15–64) [5]. This is a 23% increase, partly due to population growth, with approximately 35 million of the people who used drugs (0.7% of the adult population) having substance use disorders that could benefit from treatment [6]. Addictive disorders (behavioural addictions in ICD-10) are of similar concern, with global estimates of 0.12–5.80% for gambling disorder [7] and 1.96–3.05% meeting the threshold for the recently recognised internet gaming disorder in the International Classification of Diseases (11th ed) among the almost 3 billion gamers worldwide [8]. However, many countries have a limited response to these conditions and carry negative and stigmatized approaches to the needs of such people in need of treatment. The global scale of these problems, the limited access and late entry to treatment, and the accompanying high level of stigmatization make accessible and effective intervention a critical challenge for health and care systems in the coming decade.

Current treatment options for people with substance use and addictive disorders include psychosocial and pharmacological interventions. Interventions may be delivered across a range of health care contexts, including inpatient, residential, outpatient, and virtual settings, as well as in criminal justice, education, and social care environments. This complexity is further exacerbated by geographical variation in practice as well as the fact that people with substance use or addictive problems often access multiple services [9,10,11,12]. This makes disentangling what genuinely works, and for whom, challenging. Finding consensus in partnership with people with lived experience over the priority outcomes, and measuring these outcomes in a meaningful and standardised manner across the continuum of healthcare, is a necessary first step.

Routine outcome measurement can be challenging in practice [13], as defining, agreeing, and measuring outcomes can be difficult due to the considerable variability in the tools used to measure outcomes and difference in interpretations for the ultimate goal of treatment [14,15]. Numerous efforts exist to standardise outcome measurements for substance use disorders and high-risk substance use; for example, there is an international consensus set for alcohol brief interventions designed for adult non-treatment-seeking populations which may not be suitable for those actively seeking treatment [16,17]. Others exist for gambling treatment [18] specific drugs [19], and alcohol and drugs more broadly [20,21]. A comprehensive outcome set designed for assessment of substance-related and addictive disorders could support longitudinal analyses, especially when considering capture of sensitivity to change across addictions. Those with addictions may present complex profiles of multimorbidity that require a broader set that is better placed to capture this heterogeneity.

Accurately measuring the impacts of treatment ensures that the most effective approaches are developed and integrated across health and social systems. Future developments need availability of frameworks and outcome measures to assess the value and quantify the impact of treatment, to support appropriate interventions. National programs have developed “outcome monitoring systems” to collect data and provide information on the effectiveness of national treatment programs. However, harmonisation of national data programs remains elusive and ambitious, and the rise of various behavioural addictions requires further adjustments to measures. Recent work on patient-reported outcome measures (PROMs) has made inroads to this challenge and ongoing developments in Health Informatics make it important to reach agreement on measurements in the addictions field [22].

Common Data Elements by the Clinical Trials Network (NIDA) and NIDA Phenx are examples of consensus-based standard sets developed to provide broad outcome measurement and improve data harmonisation across populations who use substances [23,24]. While these sets are effective in supporting interoperability and standardising recording they were developed as efforts to identify and measure common data elements for health record systems and clinical research; involving the screening, brief intervention, and referral to treatment for substance use disorders [25,26].

In recognition of the value of building consensus for common approaches to outcome measurement, the International Consortium for Health Outcomes Measurement (ICHOM), a non-profit organization that leads efforts to develop minimum “Sets of Patient-Centered Outcome Measures” that matter to people across a broad range of health conditions, took the initiative to develop a set of routine measures for addiction treatment services. Sets are created around conditions, or groups of conditions, which might have similar outcomes of importance. They are developed by international experts convened by ICHOM and that cover a range of expertise. The recommendations include the outcome domains that are important conceptually as well as a recommendation of possible tools that exist to optimally capture these. Recommendations are derived from a review of the existing scientific literature (validation studies, systematic reviews) and structured discussion among convened experts, balancing several pragmatic considerations. These include the cost of administering tools, the psychometric evidence available to support their use, their international applicability, and whether the tools can appropriately cover the concepts of interest whilst minimising the burden for people completing them.

This manuscript reports the process of developing a such a set in the field of addictions. Principles and aims of the set were to (a) capture the most important patient-centred outcomes, (b) be applicable in standard clinical practice, and (c) be acceptable to health care providers and patients across contexts and cultures. It is not the aim of this exercise to develop gold standard instruments but more simply to build consensus to enable broader consistency of measurement of treatment outcomes. Consistency across measurement will enable comparative research to investigate outcomes across varying treatment settings, modalities, and populations leading to the generation of research identifying the best available treatments. By providing policy makers, service providers, and healthcare professionals with information on best practice, this set aims to improve healthcare standards and outcomes for those who access these services. Ideally, this is an iterative living process where the focus of the measures is continually refined and focused to capture key outcomes as brief and time efficiently as possible.

In this document, we will firstly describe the process and procedures of each consensus phase: (1) a modified Delphi process and (2) an open review. Through this process we will identify (1) outcome domains, (2) outcome measures, (3) case-mix variables that moderate and enhance understanding of treatment outcomes, and (4) suggested timepoints for treatment evaluation.

## 2. Materials and Methods

### 2.1. Project Team

The project team was led by the chair (MF), a research fellow (NC), and two project managers (SC and LSF). This team facilitated the consensus process by performing systematic and scoping reviews, preparing evidenced-based tables based on these reviews, making recommendations based on these evidence-tables, and raising key questions in advance, which were all presented to the working group across a series of videoconferences via Webex (Version 2018-12-02.65ab2d5). Project team members did not participate in voting. This work was registered and completed in accordance with methodological recommendations by the Core Outcome Measures in the Effectiveness Trials (COMET) Initiative [27], COMET number 1185.

### 2.2. Working Group

The project followed a structured, consensus-seeking process with all key decisions voted on by a quorum number of working group members, and a set threshold of consensus (70–80% depending on the decision stage). The work was undertaken as a series of videoconferences in which working group members discussed all key elements of the set. These videoconferences were followed by iterative anonymous surveys and a Delphi procedure to achieve consensus [28].

The working group (co-authors 5–30) was convened by ICHOM and comprised 26 experts from 11 countries across 5 continents. Working group members were carefully selected to ensure the overall group covered diversity in expertise (across lived experience, clinical practice, and the professional disciplines listed below) and geographical location. Almost half (44.8%) of the working group had clinical experience, with an average of 26.54 years (*SD* = 10.84, Range: 3–40 years) of experience. Over half (62.1%) had research expertise in substance use disorders and almost a third (34.5%) in behavioural addictions, with an average 25.20 years (*SD* = 9.09, Range: 9–46) and 24.60 years (*SD* = 8.26, Range: 9–35) of experience in each expertise, respectively. There were 5 (19%) individuals with lived experience expertise in the working group, and under half (48.3%) of the working group identified any prior involvement in outcome measurement and/or measurement tools, including the development, experience, validation, evaluation or other interest in any measurement tool. To avoid institutional biases, only one working group member was permitted from any given department of an institution, and experts participated in their personal capacity, not as representatives of any group or entity. While the Australian context had higher representation among panel members and greater heterogeneity is preferrable for a working group, this overrepresentation of Australian experts does not necessarily compromise the appropriateness of the panel for reaching consensus [29]. Five working group members (19%) were recruited to the working group as lived experience experts. Over half of the working group were senior clinical researchers with extensive publications on treatment evaluation and treatment outcome measurement, including psychometric knowledge and experience. Of the remaining members, most had professional experience across psychology, psychiatry, and social work. Working group members also had professional expertise in public health and epidemiology, mental health nursing, evidence-based addiction medicine, substance use disorder epidemiology and biostatistics, health economics, primary care, and patient-reported outcome measures. Eight (31%) working group members were currently practicing (e.g., medicine, social work, psychology). Additionally, 12 (46%) working group members were female, and 14 (54%) were male. All working group members volunteered their time without financial compensation and had an equal vote on every element of the set development process.

### 2.3. Procedure

#### 2.3.1. Modified Delphi Process

**Figure 1:**
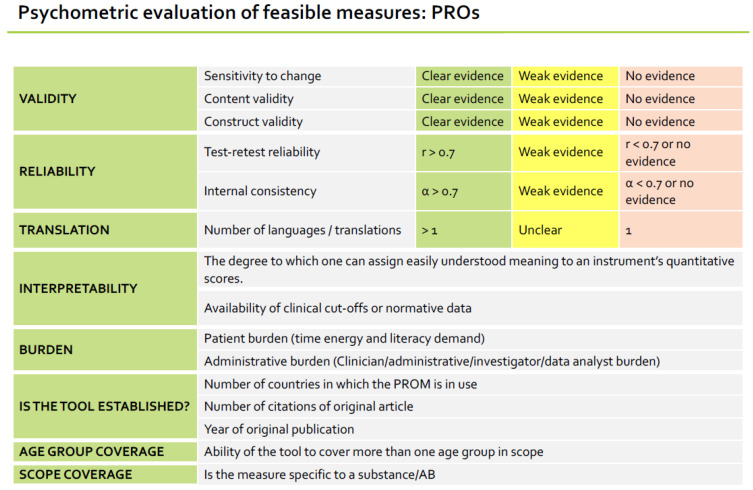
Format of “Psychometric evaluation of feasible measures” as displayed to working group members.

Each videoconference was preceded by extensive preparatory research conducted by the project team, which is summarized in Figure 1 and described throughout the Supplementary S1: Additional methodological information. This research involved systematic reviews, advisory groups, and reviews of supplemental sources to develop an exhaustive list of potential outcome domains, outcome measures, and case-mix factors. All identified outcome domains and case-mix factors were extracted, synthesised, and collapsed into meaningfully indistinct groupings. Outcome measures under consideration were reduced through two steps before presentation to the working group. First, a feasibility assessment: whether an instrument was free to use in clinical practice, captured the outcome domain, took less than 20 min to complete, and had evidence of psychometric quality. Second, a detailed psychometric review was undertaken in line with International Society for Quality of Life Research guidelines [30], including reliability (test–retest, internal consistency), validity (content, construct, sensitivity to change), ease of interpretation, burden, translations available, and coverage of scope (i.e., relevant disorders and age groups). A summary of how the selected measures performed on each criterion is provided in Table 1.

This information was presented to the working group during the videoconferences to inform their decisions, see Figure 1.

An example of how the psychometric properties of generic measures of substance use were presented for consideration to working group members is shown in Table 2.

Each videoconference ran for 90 min and was undertaken twice to facilitate different time zones across the globe. Videoconferences consisted of presentations relating to background research, rationales, proposals or options, and working group member comments from previous votes, which guided a targeted discussion focusing on key questions or elements crucial at each stage of the set development. PowerPoint slides were sent to working group members a week ahead of each call to allow time for preparation.

Following each call, minutes of both meetings (in the two time zones) were circulated, and the working group members completed a survey to vote and comment on the key decision points raised during the videoconference. In deciding on the outcome domains, a modified Delphi procedure rather than a single survey was used, with comments of the working group members shared anonymously. An 80% working group response rate was minimally required for a survey to be considered valid. A 70% consensus level was minimally required for all decisions except the outcome domains, which required minimally an 80% consensus on the first two rounds and minimally 70% on the final round. The higher threshold for the first round of outcome domains was selected to reflect the importance of this decision to the set overall and because it is possible to use a higher threshold given that Delphi processes are iterative. If agreement was not reached the project team refined the proposal to incorporate working group feedback and a second round of votes was taken with working group members’ free-text responses shared anonymously with the full working group to facilitate discussion.

#### 2.3.2. Open Review

Once all decisions were made, an open international consultation of professionals and people with lived experience was conducted to receive feedback on the set recommendations. The lived experience survey was limited to countries in which ethics approval was granted or waived (UK and USA), whereas the professional survey was open to people in all countries. Finally, the project teams for all ongoing mental health sets had a discussion to identify areas for potential harmonisation across the sets. For example, consensus was sought for case-mix variables to be included and developed with a harmonised definition in tandem with the ICHOM Mental Health Set. Achieving harmonisation across various sets aims to facilitate the implementation of these sets into systems while working towards the iteratively cohesive measurement of outcomes. The outcome of this was discussed with the working group for final decision making. A full overview of the supporting research is elaborated upon in Supplementary S1: Additional methodological information.

## 3. Results

### 3.1. Recommended Outcomes and Measures

#### 3.1.1. Population

Building on existing standardisation efforts within addiction research and treatment, the working group sought to recommend a set of measures applicable across disorders and settings and incorporating not just alcohol and other substance use disorders, but also tobacco, gambling, and gaming disorders.

The scope for the set includes patients aged 12 years or older in any addiction treatment setting or delivery method. This lower age limit was recommended by the working group as the burden of disease, age of onset, and youngest treated samples for substance use and behavioural addictions are typically from 12 to 15 years old. Similarly, outcomes important in childhood may be developmentally specific and require separate consideration from adults. Included are harmful patterns of use, abuse, dependence, and withdrawal of alcohol, tobacco, and other drugs (excluding caffeine, which was excluded from the scope via consensus vote), and gambling and gaming disorder. Disorders of a single episode of harmful use, intoxication, substance induced delirium, and substance-induced psychosis were excluded by consensus vote because the clinical features, treatments, and outcomes of these disorders were judged to be markedly different for the target disorders.

#### 3.1.2. Outcome Domains

**Figure 2:**
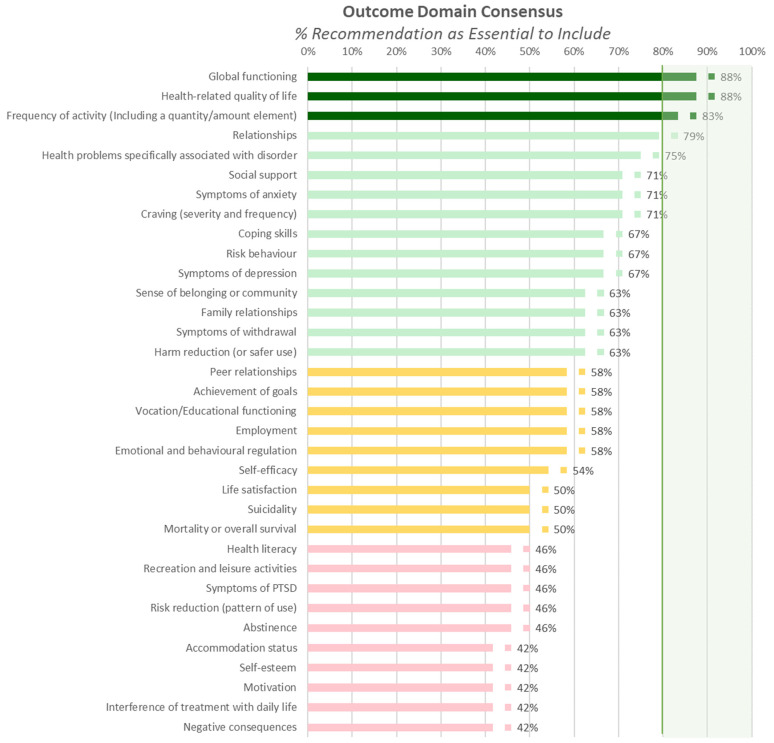
Percentage of working group (*n* = 24) that recommended an outcome domain as essential; for consensus of >40%. 80% consensus for essential recommendations (dark green), 50–79% for further discussion and potential inclusion (60–79%, light green; 50–60%, yellow) for and exclusion (<50%, red). See Supplementary S2 and S3 for more detail on voting and outcome domains less than 40%.

In total, 80 possible outcome domains were identified as part of the systematic review process. Two voting rounds for outcome domains were conducted, the first round of voting is shown in Supplementary S2. Working group members voted for recommendation of each outcome domain on a scale from “1—Not Recommended” to “9—Essential to Have”, with votes from 7 to 9 considered as endorsing recommendation. Domains recommended by 50–79% of the working group in voting round one (failing to meet 80% recommendation) were included in a second round of voting shown in Supplementary S3. Consensus was met to exclude all domains that were recommended by less than 50% of the working group in round one. Of the 80 outcome domains considered, seven were voted in for inclusion (each with 83–92% endorsement). Outcome domains recommended as essential by at least 40% of the working group are shown in Figure 2.

Of the seven outcome domains, two domains related to the addictive disorder (frequency and quantity of addictive behaviour and symptom burden) and five domains related to overall functioning and wellbeing (health-related quality of life; global functioning; overall physical health and wellbeing; overall mental health and wellbeing; and psychosocial functioning) were included. These outcome domains, as well as the corresponding measures recommended by consensus voting, are summarised in Figure 2.

#### 3.1.3. Outcome Measures

Currently no parsimonious measurement tools capture outcomes above and beyond all others without shortcomings or limitation. The existing psychometric evidence base for substance use and, to a far greater degree, addictive behaviours (gambling, gaming), present insufficient evidence to have a set that is currently fit for the purpose. While the working group had no obvious pathway to discerning recommendations, this work represents an attempt to raise these issues, make an initial recommendation, and progress the addiction field forward.

Overall, 158 measures were initially available for consideration to the group, with some additional measures being suggested during videoconferences as relevant. These were mapped to one or more of the seven recommended outcome domains. Mapping was undertaken based on the initial construct the measure was developed to capture, as articulated in its development paper. Decision-making for inclusion and/or exclusion for all considered measures is detailed along with primary reasonings for exclusion in Supplementary S4. Numerous measures did not meet criteria when assessed for feasibility and were excluded before psychometric appraisal.

To assess frequency and quantity, the Treatment Outcome Profile (TOP) [20] was recommended, with additional items to capture tobacco use (as per the Australian TOP) [21], gambling, and gaming. This was based on the brevity of the TOP, that it was free to access and use, and had sufficient psychometric evidence and permission for translation. The TOP is based on timeline follow-back methodology and was recommended due to its comparatively low burden and ability to break down responses by weeks. Its sensitivity to change was limited but similar to that of other instruments. With 21 items, the proposed modified TOP with additional items is one of the longest measures proposed for inclusion, but this was justified by the fact that the measure captures frequency and quantity for all included addictive substances and behaviours. It is proposed that this modified TOP be measured for all patients. Adaptive logic is available such that if the patient indicates no gambling, then subsequent items on engagement with individual gambling products need not be assessed. The other reason for including the TOP was because it has been used as a National Outcome Measurement tool in the English National Health Service since 2008, and an adapted version has been implemented in Services in New South Wales, Australia and increasingly in other parts of Australia. Evaluations of psychometric properties presented to the working group throughout the consensus process are shown for the TOP, see Table 3.

To assess symptom burden, the Patient-Reported Outcomes Measurement Information System (PROMIS) measures were recommended for alcohol-, drug-, and tobacco-use-related symptoms [31,32,33]. The Problem Gambling Severity Index (PGSI) [34] was recommended for gambling-related symptoms, and the Internet Gaming Disorder Test (IGDT-10) [35] was recommended for gaming-related symptoms despite these measures being less robustly evaluated. Completion of these symptom burden measures is proposed as conditional on the patient experiencing a problem with a given substance or behaviour. The PROMIS measures have been primarily validated in US samples. However, they are short, with valid coverage of the outcome domains and have been rigorously developed in consultation with people with relevant lived experience. Furthermore, for tobacco, one additional item from the two-item Heaviness of Smoking Index (HSI) [36] was also recommended (time to first cigarette) to increase comparability with existing work. The other HSI item, cigarettes smoked per day, is captured by the modified TOP, completing the measurement of the full HSI in this set. The PGSI and IGDT-10 were considered the best available options; note however, they were developed as screening measures to detect problems and have limited validation in detecting change in a treatment context.

To assess the five domains of functioning, in respect of the largely overlapping nature of these domains, the working group first attempted to identify a single all-inclusive measure to minimize patient burden. When evaluating the existing measures, a key distinction was apparent between generic measures (not disorder-specific and therefore allowing comparisons across disorders) and those specific to disorders related to substance use and addictive behaviours (therefore capturing the elements of functioning that are important especially for populations experiencing problems in these areas). The working group decided to include one generic and one specific measure to improve the utility of the set.

**Table 3:** Summary of validity, reliability, and clinical utility of measures recommended by consensus to be included in the set.

		Outcomes	Validity ^3^				Reliability ^4^	
Areas ^1^ *	Measure	Primary Domain ^2^	Content	Construct	Change *	Countries with Validation Studies *	Test–Retest	Internal *
All Disorders *	Treatment Outcome Profile (TOP), with recommended additions	FQ				Australia, Chile, China, UK		
*Alcohol*	PROMIS Alcohol Use 7a	SB				USA		
*Drugs*	PROMIS Severity of Substance Use past 30 days 7a	SB				USA		
*Smoking*	PROMIS Nicotine Dependence for All Smokers 8a	SB				USA		
*Smoking–1-item*	Heaviness of Smoking Index	SB				Australia, Brazil, Canada, France, Germany, Malaysia, Spain, Switzerland, Taiwan, UK, USA		
*Gambling*	Problem Gambling Severity Index (PGSI)	SB				Australia, Canada, Italy, South Korea, Spain, Sweden, Taiwan, USA		
*Gaming*	Internet Gaming Disorder Test (IGDT-10)	SB				Australia, Belgium, Canada, Czech Republic, Finland, France, Hungary, Iran, Italy, Norway, Peru, Slovakia, Slovenia, South Korea, Taiwan, Turkey, UK, USA		
*GF:Adults and* *Adolescents*	World Health Organisation Disability Assessment Schedule (WHODAS)	QoL				Canada, China, Poland, Rwanda, “International” [35]		
*GF:* *Adolescents*	KIDSCREEN-10 for services that exclusively treat adolescents	QoL				Austria, Belgium, Bulgaria, Czech Republic, France, Germany, Greece, Greenland, Hungary, Iran, Ireland, Japan, Luxembourg, Macedonia, The Netherlands, Poland, Portugal, Romania, Russia, Slovenia, Spain, Sweden, Switzerland, Turkey, UK		
Disorder specific *	Substance Use Recovery Evaluator (SURE)	QoL				UK		
Physical Health *	1 item from PROMIS Global Health: “In general, how would you rate your physical health?”	PH				Brazil, USA		
Mental Health *	2 items from PROMIS Global Health: “In general, how would you rate your mental health, including your mood and your ability to think?”; “How often have you been bothered by emotional problems such as feeling anxious, depressed or irritable?”	MH				Brazil, USA		
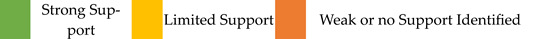								

Areas ^1^: All Disorders *: alcohol, drugs, smoking, gaming, gambling; “GF: Adults and Adolescents”: generic functioning for services treating adults and services that follow adolescents into adulthood; “GF: Adolescents”: generic functioning: for services that exclusively treat adolescents; “Disorder specific *”: disorder specific: also administered if problem relates to alcohol or drugs. Physical Health *: overall physical health and wellbeing; Mental Health *: overall mental health and wellbeing. Outcome domains ^2^: FQ: frequency and quantity; SB: symptom burden; QoL: health-related quality of life, global functioning, and psychosocial functioning; PH: overall physical health; MH: overall mental health. Validity ^3^ “Change *”: sensitivity to change. Countries with Validation Studies *: where consensus process is undertaken. Reliability ^4^: “Internal *”: internal consistency.

The Substance Use Recovery Evaluator (SURE) [37] was recommended as the specific measure for problems related to alcohol and drugs; no suitable specific functioning measures were identified for problems related to tobacco, gambling, and gaming. The SURE is a relatively new measure and at the time of voting had only been validated in the UK, with limited information on sensitivity to change. The other considered measure—the Short Inventory of Problems: Alcohol and Drugs (SIP-AD)—similarly had limited information on sensitivity to change as well as on test-retest reliability. The working group, including those with lived experience, considered the SURE to be the preferred option because it was based on the perspectives of people with lived experience and covered condition-specific outcomes important to this group. The SURE was seen to capture domains identified by members with lived experience as important (namely, acceptance and understanding, coping, relationships and social support, and control and normalcy). The SURE also covers substance use, self-care, material resources, and outlook on life. Meanwhile, German, French, and Dutch language versions of the SURE are available.

Initially, the working group recommended the PROMIS Global Health as the generic functioning measure. However, following harmonization discussions across the ICHOM teams conducting the mental health sets, the working group on request from the mental health team changed their recommendation to the most popular option across the different disorder sets. This was the World Health Organization Disability Assessment Schedule (WHODAS) [38] for all services treating adults and services that follow adolescents into adulthood and the KIDSCREEN-10 [39] for all services that exclusively treat adolescents. This means that service users accessing treatment for multiple mental disorders would only need to complete one global functioning measure. Moreover, the WHODAS permits calculation of quality-adjusted life years (QALYs) for the purpose of economic evaluations. Since the WHODAS and the KIDSCREEN do not capture all functioning domains included in this set, the working group proposed that additional single items from PROMIS Global Health should be included to assess overall physical and mental health and wellbeing.

### 3.2. Recommended Case-Mix Factors

The working group agreed upon a minimum set of 14 case-mix variables to be measured at baseline. These are characteristics that allow for meaningful comparisons of treatment outcomes between settings based on demographic and clinical predictors of outcomes (see Table 4). Decision-making for case-mix variables is shown in Supplementary S5.

### 3.3. Recommended Measurement Time-Points

Longitudinal measurement of outcomes is critical to assessing meaningful change over time. To encourage this, the working group proposed all outcome measures be collected at baseline (intake or within one week of intake), throughout active treatment (at least every three months), at discharge or transition to another level of care, and annually for two years following the end of active treatment. There was a realistic understanding that most services would collect data during service contact and not beyond that, but those with lived experience expressed the view that longer-term follow-up assessments reflected the working group’s belief that recovery from substance use and addictive disorders take time and that relapse is always a possibility. Ideally future national data linkage programs could provide data on longer term outcomes and other forms of service contact and utilization.

## 4. Discussion

We developed a minimum set of patient-reported outcome measures (PROMs) for people seeking treatment for an addictive disorder to provide a common standard for routine outcome measurement. This represents one of the first initiative of its kind to incorporate additional addictive disorders alongside substance use disorders. Over the course of 16 months, an international, multidisciplinary working group of 26 experts—including professionals and people with lived experience—worked to agree on the elements most important to include in a minimum set. The result is a parsimonious set that balances the priorities of different disciplines and disorders with the need to reduce, to the extent possible, administrative burden in clinical practice. We hope that standardized measurement of treatment outcomes, using this globally applicable set, will support professionals and patients and lead to greater value in healthcare. In the future, we hope it will enable available more effective funding allocation and the synthesis of high-quality evidence on what works, and for whom. At the local level, the set might serve as a case management tool, directly contributing to and improving the patient experience as part of measurement-based care although, further evaluation for this purpose is needed. The set can be used by clinicians and practitioners treating people with addiction related problems, including physicians, nurses, social workers, and psychologists. The set could also be used in clinical trials to assess the impact of novel treatments on these important outcomes and by treatment commissioners to set measurable goals, assess the quality of treatment services, and support system-level performance measurement.

### 4.1. Strengths of the Set

This set is a first proposal, based on existing measures, the accompanying available evidence, and international expert opinion, including people with lived experience. While the individual measures included in the set have been validated, validation across all possible settings, specific populations, measurement consistency cross-culturally, and the set in its entirety requires further efforts and data pooling for evaluation. For this purpose, a steering committee comprised of a subset of volunteer working group members has been established to support the continued development of the set. Meanwhile, a prospective study with the set has been initiated in Belgium, and the first results and experiences will be available soon [40]. We actively encourage those who implement the set to provide feedback to the steering committee via ICHOM to facilitate improvements and updated proposals. This will be a crucial step in ensuring that the set meets the continued needs of treatment providers and those seeking treatment.

### 4.2. Limitations and Areas of Future Research

The broad range of existing measurement tools across the addiction field is a key source of research waste and contributes to unnecessary knowledge gaps; necessitating the development of core outcome sets that standardise measurement, synthesise evidence, and measure outcomes that matter [15]. Different organizational settings have adopted a broad and eclectic range of measures, many of which have been validated for the intended purpose. It was not the purpose of this exercise to assess or confirm the superiority of one instrument over others. The purpose of the working group was to be guided by utility and availability of measures and to achieve consensus on the current choice, creating the best possible priority measures available at the time. The instruments still carry a time burden for completion and will require further development and refinement and adaptation for electronic data collection and storing of this information. Ideally, in the future this would be built into routine clinical practice. This record would then be used both for clinician and service user to assess the impact of treatment. The principles of brevity, accuracy, free public availability of measures, multiple validated language versions and sensitive measures of change will require ongoing work to ensure all common measures exceed these criteria. The use of this set will help guide future revisions.

The scientific literature on gambling and gaming problems is relatively new compared to substance use and, hence, some challenges faced by the working group occurred in this domain. In defining the scope of the set, the working group decided to include two behavioural addictions already included in the ICD-11 (gambling and gaming). Other addictive behaviours might be included in the future as the validity of the concepts and their link with disorders related to substance use, gambling, and gaming increases.

We encourage further validation research for gambling and gaming outcome measures. The included screening measures might be replaced by newer, brief, outcome measures as the evidence for their validation becomes stronger. Our proposal to add quantity–frequency items for gambling and gaming into the existing TOP measure requires validation. No suitable condition-specific functioning measures were reviewed for gambling and gaming (or tobacco), highlighting a need for further development of brief validated measures in these domains. We ran each videoconference twice, allowing us to accommodate all global time zones. However, this meant that all working group members did not get to discuss the proposals synchronously. We attempted to mitigate this by using identical call materials and facilitators each time and sharing any highlights from the preceding with the subsequent call. Minutes of both calls were combined and shared with all members before decisions were made via a post-call vote, which was undertaken and reported collectively.

Global functioning was recommended as an essential outcome by the working group but identifying measures that captured aspects relevant to people with lived experience was challenging. Many measures did not capture the consensus of content identified by members of the working group, including those with lived experience. As the conceptualization of quality of life and functioning was critical to the working group’s consensus process, the SURE was recommended to measure global functioning despite limited psychometric evidence compared with other measures such as the SIP-AD. This recommendation is intended as a launching point for further psychometric investigation when considering the measurement of global functioning, particularly to encourage investigations aimed at improving the face and content validity of such tools.

The conflation between screening tools and outcome measures is common and consequential in the addiction field [41]. In selecting outcome measures for symptom burden, the most fit-for-purpose measures identified were screening tools. Such measures focus on identifying the disorder and not tracking outcomes and as such might be limited in their ability to detect meaningful change, especially at lower levels of severity. For example, they often contain broad timeframes for recall (e.g., “ever in your lifetime”, “in the past year”), are sensitive to less severe symptoms, and—if particularly sensitive (rather than specific)—lack accuracy and range for capturing symptom severity in specific populations, often being employed for general populations. As such, screening tools can be insufficient for capturing and evaluating the outcomes of addiction treatment. As an example, the AUDIT was regarded unsuitable for the set since the “past year” timeframe made this measure insensitive to short-term changes in alcohol dependence severity during treatment.

However, sensitivity to change is not a limitation unique to screening tools and was seen as a major limitation across all measures. This issue extends beyond the development of sets and is more broadly seen in addiction research and clinical practice, which currently lack a gold standard for assessing sensitivity to change. All included measures throughout this document were consistently scored by the working group as having weak sensitivity to change, exemplifying this issue. While no measure effectively capturing sensitivity to change is currently supported, the recommendations made in this document aim to move the field forward, highlight current shortcomings, and subsequently facilitate the improvement of capture for such factors.

The working group aimed to strengthen international capacity by pooling expert members with international outcome measures experience, resulting in a lower representation of practicing clinicians. As such, many members of the working group have been instrumental in supporting initiatives aimed at the development and evaluation of various outcome measures. By declaring potential conflicts of interest, running a structured consensus process, and requiring a high consensus threshold for recommendations, we aimed to mitigate any risk of resulting bias from members with extensive impact in the outcome measure space. The external consultation also served as an opportunity to bring in a greater diversity of views and voices. However, the open review process received limited input and primarily garnered respondents from high-income countries, resulting in a lack of representation outside of this context, limiting global applicability. Despite the inclusion of perspectives from people with lived experience, there was still limited ability to fully represent the addiction field which comprises of diverse backgrounds and complex presentations. Sample diversity was not considered when appraising psychometric evidence or validation papers for specific tools which, despite limited evidence existing, is still a limitation of the current document.

### 4.3. Implementation

To encourage and enable uptake and implementation of the set, a full reference guide and data dictionary are available for free upon registration via the ICHOM website: https://connect.ichom.org/standard-sets/disorders-related-to-substance-abuse-or-addictive-behaviours/, accessed on 21 February 2024.

There is a growing community of healthcare providers implementing the ICHOM sets, which have been demonstrated to be feasible [42]. The ICHOM implementation framework outlines a structured process to guide implementation for organizations wishing to collect routine outcome data. Typically, an implementation project takes 9 months to complete. This includes the translation of questionnaires, assessing IT and informatics infrastructure, performing a gap analysis of data already collected, assessing and defining scope for the project, securing measure licenses as required, determining and process-mapping the implementation site, recruiting a local project manager, and securing additional information platforms to address data gaps.

To date, a research group in the Netherlands and Belgium has undertaken work to demonstrate that it is feasible to implement this within a structured service evaluation and that further work is underway to refine this work.

Implementation of routine outcome measurement may be challenging for treatment centres and systems as the proposed set can be a substantial time burden over current measurement practice. In the Belgium work, the average time to complete the battery of instruments was 40 min. In Ontario, Canada, the set has provided the basis for the selection of common outcome domains being implemented across six diverse, bed-based substance use treatment sites with the initial research goals focused on the development and evaluation of the implementation process itself [43,44], drawing upon an implementation science framework [45]. To highlight eventual use of the results, key lessons learned include the importance of providing implementation supports such as technical advice on measurement-based care, staff training and competency, organizational commitment and leadership, and synchronicity with on-site information technology [44,46].

Challenges in implementation notwithstanding, the increasing demand for implementation of quality standards also requires standardization of outcomes measures [6]. The recent experience with the COVID-19 emergency measures brought more addiction services to use some level of digital interventions. Where feasible, electronic measurement and linking with electronic medical records can reduce the paperwork, by automatizing some elements for program staff if patients are able to input their responses directly into the system. We encourage those who implement the set to report back on the metrics associated with its use in their service.

Given the goal of developing a parsimonious set, we recognize that the proposed set may inevitably fail to capture all outcomes that matter across diverse treatment settings, for all included disorders, or to individuals in all cultural settings around the world or within all subpopulations (e.g., those experiencing homelessness). This recommendation is considered a minimum, and individual treatment settings are encouraged to use their own judgment regarding which other outcomes are important to measure in their setting, in addition to those in the proposed set. The work completed in this document reflects the evidence available during consensus proceedings. As addiction policies, interventions, and relevancies develop dynamically across time, it is vital to continue providing recommendations based on the highest-quality and most recent evidence. This work creates a platform for continuing efforts aimed at improving outcome measurement and practice in the rapidly evolving addictions field.

## 5. Conclusions and Call to Action

We outline the first global effort to standardize outcome measurement across both substance-related disorders (alcohol, drugs, smoking) and addictive disorders (gambling, gaming) together. The expert working group reached consensus for essential outcome domains, recommending frequency and quantity of addictive disorders, symptom burden, health-related quality of life, global functioning, psychosocial functioning, and overall physical and mental health and wellbeing. To measure these domains, the working group developed consensus for a minimum set of patient-centred outcome measures that can be implemented across treatment settings for substance-related and addictive disorders globally. Implementation of this person-centred set will facilitate comparative research and better enable comparisons across disorders and treatment settings and modalities, providing patients, professionals, managers and policy makers with the information about the best possible treatments, and driving improvements in value in healthcare. Measurement practice requires continuous evaluation for relevance and utility across contexts and time.

## Figures and Tables

**Table 1:** Measures selected through consensus recommendation across outcome-domains recommended as essential for alcohol, drugs, tobacco, gambling and gaming.

	Alcohol	Drugs	Tobacco	Gambling	Gaming
Frequency and quantity	Treatment Outcome Profile (TOP), with additions				
Symptom burden	PROMIS Alcohol Use 7a	PROMIS Severity of Substance Use past 30 days 7a	PROMIS Nicotine Dependence for All Smokers 8a and Heaviness of Smoking Index	Problem Gambling Severity Index (PGSI)	Internet Gaming Disorder Test (IGDT-10)
Health related quality of life	Generic: World Health Organisation Disability Assessment Schedule (WHODAS) for services treating adults and services that follow adolescents into adulthood; KIDSCREEN-10 for services that exclusively treat adolescents Condition specific: Also administer the Substance Use Recovery Evaluator (SURE) if problem relates to alcohol or drugs				
Global functioning					
Psychosocial functioning					
Overall physical health and wellbeing	Single item from PROMIS Global Health: “In general, how would you rate your physical health?”				
Overall mental health and wellbeing	Two items from PROMIS Global Health: “In general, how would you rate your mental health, including your mood and your ability to think?”; “How often have you been bothered by emotional problems such as feeling anxious, depressed or irritable?”				

**Table 2:** Example of psychometric evaluation shown to working group for the “Evaluation of psychometric properties: *Generic Measures*” of substance use measures.

	Sub-Criterion	SF-36	EQ5D	WHO QOL	WHO DAS-12	PROMIS Profile	PROMIS GH
**Validitiy**	Substance use and/or addictive behaviours	Drugs, Alcohol	Drugs, Alcohol	Drugs, Alcohol	Drugs, Alcohol	Some items	No
	Sensitivity to change						
	Content validity						
	Construct validity						
**Reliability**	Test–retest						
	Internal consistency						
**Translation**	Number available	>170	>170	>9	>47	>2	>2
**Interpretability**	Response scale	Mix	3 or 5	5	4	5	5
	Recall	7 or 30 days	Today	14 days	30 days	7 days	General/7 days
**Burden**	Items	20–36	6	26	12	29–57	10
**Tool** **established?**	Citations	3901	2939	3333	618	125	125
	Year published	1992	1990	1998	2010	2005	2004
**Reporters**	Self/Interview	Self	Self	Self	Self	Self	Self
	Age Coverage	Validated 13+	Youth version (12+)	Older adult version, validated to 11+	Older adult version, validated to 11+	Older adult version, validated to 11+	Older adult version, validated to 11+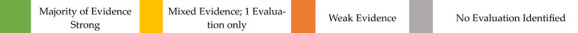							

**Table 4:** Definitions and proposed measurement of case-mix variables.

Category	Variable	Definition	Source
Demographic	Age	Year of birth	Self-report
	Sex	“What sex were you assigned at birth?” [ ] Male [ ] Female	Self-report
	Gender identity	“Do you identify yourself as…?” [ ] Boy/Man [ ] Girl/Woman [ ] Non-binary [ ] Trans man/Transgender man/FTM [ ] Trans woman/Transgender woman/MTF [ ] None of these describe me, and I’d like to specify_________ [ ] Prefer not to answer	Self-report
	Sexual orientation	“Do you identify yourself as…?” [ ] Straight or heterosexual [ ] Gay or lesbian or homosexual [ ] Bisexual [ ] None of these describe me, and I’d like to specify_________ [ ] I don’t know right now [ ] Prefer not to answer	Self-report
	Socioeconomic status	Adults: highest level of education completed. Adolescents: proxy to be used: highest level of education completed by parents.	Self-report
	Work status	[ ] “What is your work status?” [ ] Unable to work [ ] Not working by choice (retired, homemaker) [ ] Seeking employment (I consider myself able to work but cannot find a job) [ ] Part-time work, school, or vocational training [ ] Full-time work, school, or vocational training	Self-report
	Accommodation or homelessness status	SURE: “I have had stable housing” [Past week] Treatment Outcome Profile/Clinician report: “At risk of eviction” [Yes/No] “Acute housing problem” [Yes/No]	Self- and clinician-report
Clinical	Genetic disposition	“Have either of your biological parents or siblings had an alcohol problem?” [Yes/No/Don’t know] “Have either of your biological parents or siblings used non-prescribed drugs?” [Yes/No/Don’t know]	Self-report
	Environmental exposure	“Do you live with anyone who has a current alcohol problem?” [Yes/No] “Do you live with anyone who currently uses non-prescribed drugs?” [Yes/No]	Self-report
	Exposure to negative life events and their impact	Primary Care PTSD Screen (PC-PTSD-5) Sometimes things happen to people that are unusually or especially frightening, horrible, or traumatic. For example, a serious accident or fire; a physical or sexual assault or abuse; an earthquake or flood; a war; seeing someone be killed or seriously injured; having a loved one die through homicide or suicide. 1. Have you ever experienced this kind of event? [Yes/No] If no, screen total = 0. Please stop here. If yes, please answer the questions below. In the past month, have you… 2. had nightmares about the event(s) or thought about the event(s) when you did not want to? [Yes/No] 3. tried hard not to think about the event(s) or went out of your way to avoid situations that reminded you of the event(s)? [Yes/No] 4. been constantly on guard, watchful, or easily startled? [Yes/No] 5. felt numb or detached from people, activities, or your surroundings? [Yes/No] 6. felt guilty or unable to stop blaming yourself or others for the event(s) or any problems the event(s) may have caused? [Yes/No]	Self-report
Intervention	Intervention setting	“Please indicate in which setting an intervention has taken place. Please check all that apply” [ ] Residential or inpatient treatment [ ] Non-residential or outpatient treatment [ ] Day treatment [ ] Digital [ ] Other	Clinician-report
	Intervention type	“Please indicate the type of intervention. Please check all that apply” [ ] Medication for substance use (including agonist treatment) [ ] Counselling or psychotherapy [ ] Other	Clinician-report

## Data Availability

The final set of patient-centred outcome measures, as well as data dictionary and reference guide are freely available at this link: https://connect.ichom.org/patient-centered-outcome-measures/disorders-related-to-substance-abuse-or-addictive-behaviours/, accessed on 21 February 2024. Data are included in the article and Supplementary Materials as aggregated values; further inquiries can be directed to the corresponding author.

## Supplementary Materials

The following supporting information can be downloaded at: https://www.mdpi.com/article/10.3390/jcm13072154/s1, Supplementary S1: Additional methodological information. Supplementary S2: All Outcome Domains with Consensus Voting and Action for Inclusion in Next Consensus Voting Round. Supplementary S3: Second round of recommendation consensus voting for Outcome Domains that were recommended as essential by 50–79% of the working group in round one. Supplementary S4. All included measures, domain and area coverage, recommendation by working group, and primary reasoning for exclusion. Supplementary S5. All considered case-mix factors, the measure or question used (if included) or considered (if excluded), and primary reasoning for inclusion or exclusion. Further elaboration on the consensus process can be found in the attached Supplementary S1.
