# Supplementary material for: An International, Multidisciplinary Consensus Set of Patient-Centered Outcome Measures for Substance-Related and Addictive Disorders
Source: J Clin Med. 2024 Apr 8;13(7):2154. doi: 10.3390/jcm13072154 (PMC11012938; doi:10.3390/jcm13072154)
Supplement: Supplementary file 1 [file jcm-13-02154-s001.zip › jcm-2906081-supplementary.pdf]


General Supplementary for **An International, Multidisciplinary Consensus Set of Patient-Centered Outcome Measures for Substance Use and Addictive Behaviour Disorders**

Nicola Black, Sophie Chung, Calvert Tisdale, Luz Sousa Fialho, Apinun Aramrattana, Sawitri Assanangkornchai, Alex Blaszczynski, Henrietta Bowden-Jones, Wim van den Brink, Adrian Brown, Qiana L. Brown, Linda B. Cottler, Maury Elsasser, Marica Ferri, Maria Florence, Ralitza Gueorguieva, Ryan Hampton, Suzie Hudson, Peter J. Kelly, Nicholas Lintzeris, Lynette Murphy, Abhijit Nadkarni, Joanne Neale, Daniel Rosen, Hans-Jürgen Rumpf, Brian Rush, Gabriel Segal, Gillian W. Shorter, Marta Torrens, Christopher Wait, Katherine Young, Michael Farrell

---

Table of Contents

General Supplementary: S1 Additional methodological information....................................3

Systematic Review of Treatment Studies 3

Overview 3

Search Terms 3

PRISMA flowchart 8

Outcome Domains 9

Overview 9

Supplementary S2. *All Outcome Domains with Consensus Voting and Action for Inclusion in Next Consensus Voting Round* 10

Supplementary S3. *Second round of recommendation consensus voting for Outcome Domains that were recommended as essential by 50-79% of the working group in round one.* 14

List of 80 unique outcome domains considered 16

Outcome Measures 17

Overview 17

List of 158 measures mapped to one or more included outcome domain 20

Summary of validity, reliability, and clinical utility information for each measure (including available translations) 22

Supplementary S4. *All included measures, domain and area coverage, recommendation by working group, and primary reasoning for exclusion.* 25

Case-Mix Factors 36

Overview 36

Search strategy for systematic review of systematic reviews 37

PRISMA flowchart for review of systematic reviews 38

List of systematic reviews included in review of systematic reviews 39

Timepoints 40

Supplementary S5. All considered case-mix factors, the measure or question used (if included) or considered (if excluded), and primary reasoning for inclusion or exclusion. 41
Open Review 45
Overview 45
Lived experience survey 46
Professional experience survey 46

References 47

Supplementary S1: Additional methodological information

Systematic Review of Treatment Studies

Overview

To generate an exhaustive pool of potential outcome domains, outcome measures, and case-mix variables the project team conducted a systematic review of peer-reviewed treatment studies for alcohol, tobacco, other drugs, gambling, and gaming disorders. To ensure full coverage, this was supplemented by existing instrument or item banks, relevant reviews, cohort studies, validation papers, and qualitative studies, an advisory group with Working Group members with lived experience, and resources suggested by Working Group members. PsycINFO, Medline, Embase, and CINAHL were searched on December 21, 2018 for studies published in the previous five . 2154 unique records were identified, of which 10% of the titles and abstracts were double screened (by NB and SC). In accordance with AMSTAR guidance, 85% agreement was achieved before proceeding to single screening of the remaining titles and abstracts. 1047 full-text articles were assessed for eligibility, of which 653 were eligible and included. The search strategy and PRISMA flow diagram, are presented in the next section.

The 653 studies included in the systematic review were grouped into four categories according to the primary substance or behaviour being targeted: namely, alcohol, tobacco, other drugs, or gambling and gaming. An efficient data extraction procedure was followed in consideration of the fact that our primary goal was to generate a large pool of potential outcome domains and measures. Specifically, minimal data (country and specific substances or behaviours being targeted) were extracted for all studies. More extensive data were extracted on all studies within each of the four categories that described a new outcome or measure. That is, we first conducted minimal data extraction and then assessed whether the study described a new outcome or measure. If not, we proceeded to the next study. If it did, we extracted full information from this study (including sample characteristics, disorder treated, treatment type and setting, case-mix variables, outcome domains, and outcome measures).

Search Terms

PsycINFO

1 ((treatment* or treated* or posttreat* or post-treat* intervention* or intervene* or therap* or psychotherap* or program*3 or pharmacotherap* or medicat*) adj6 (Patient-reported outcome* or PRO or PROs or PROM or PROMs or outcome* or efficac* or effect* or success* or impact* or evaluat* or assess*)).ti,ab.

2 exp INTERVENTION/

3 exp TREATMENT/

4 exp Treatment Effectiveness Evaluation/ or exp Mental Health Program Evaluation/ or exp Treatment Outcomes/

5 1 or 2 or 3 or 4

6 (randomi?ed or randomly or allocated or assigned or placebo).ab.

7 (RCT or controlled trial* or control trial* or clinical trial* or clinicaltrial* or open label trial* or open trial* or randomi?ed trial* or single-blind trial* or double-blind trial* or triple-blind trial* or blind trial*).ti,ab.

8 exp Clinical Trials/

9 6 or 7 or 8

10 exp Qualitative Research/

11 (case series or (qualitative adj1 (stud* or research or design* or interview*)) or ((naturalistic or case-control) adj1 (stud* or design* or trial*))).ti,ab.

12 10 or 11

13 9 or 12

14 (gambl* adj3 (dependen* or disorder* or withdraw* or abus* or addict* or harm* or problem* or pathological or compulsive or misus* or hazardous or high-risk or risky)).m_titl.

15 ((game* or gaming*) adj3 (dependen* or disorder* or withdraw* or abus* or addict* or harm* or problem* or pathological or compulsive or misus* or hazardous or high-risk or risky)).m_titl.

16 (addictive behavio?r* or behavio?ral addict*).m_titl.

17 14 or 15 or 16

18 (smoking or smoker or smokers or tobacco or nicotine or cigarette*).m_titl.

19 ("e-cigarette*" or ecigarette* or vaping*).m_titl.

20 (alcoholic* or alcoholism).m_titl.

21 ((injur* or abus* or harm* or problem* or pathological or compulsive or misus* or hazardous) adj2 (substance* or drug* or alcohol* or drink* or hallucinogen* or cannabi* or marijuana* or LSD or mescaline* or psilocybin* or hash* or tetrahydrocannabinol* or THC or heroin* or opioid* or opiate* or opium* or codeine* or Dolophine* or methadose* or fentanyl* or actiq* or duragesic* or sublimaze* or morphine* or duramorph* or kadian* or avinza* or hydrocodone* or vicodin* or oxycodone* or oxycontin* or percocet* or percodan* or oxymorphone* or opana* or sedative* or hypnotic* or anxiolytic* or barbiturate* or amobarbital* or amytal* or pentobarbit* or nembutal* or phenobarbit* or luminal* or secobarbital* or seconal* or tuinal* or Benzylbutylbarbiturate* or Butalbital* or butisol* or thiopent* or benzodiazepine* or clonazepam* or klonopin or diazepam* or valium* or lorazepam* or ativan* or alprazolam* or xanax* or chlordiazepoxide* or librium* or temazepam* or restoril* or triazolam* or halcion* or midazolam* or nitrazepam* or "sleeping pill*" or zopiclone* or zolpidem* or methaqualone* or quaalude* or mandrax* or cocaine* or crack* or stimulant* or psychostimulant* or amphetamine* or methamphetamine* or methcathinone* or "bath salt*" or cathinone* or inhalant* or mdma or mda or ecstasy or GHB or gamma-hydroxybutyrate* or dissociative* or ketamine* or PCP or polysubstance* or polydrug*)).m_titl.

22 ((dependen* or withdraw* or addict*) adj3 (substance* or drug* or alcohol* or drink* or hallucinogen* or cannabi* or marijuana* or LSD or mescaline* or psilocybin* or hash* or tetrahydrocannabinol* or THC or heroin* or opioid* or opiate* or opium* or codeine* or Dolophine* or methadose* or fentanyl* or actiq* or duragesic* or sublimaze* or morphine* or duramorph* or kadian* or avinza* or hydrocodone* or vicodin* or oxycodone* or oxycontin* or percocet* or percodan* or oxymorphone* or opana* or sedative* or hypnotic* or anxiolytic* or barbiturate* or amobarbital* or amytal* or pentobarbit* or nembutal* or phenobarbit* or luminal* or secobarbital* or seconal* or tuinal* or Benzylbutylbarbiturate* or Butalbital* or butisol* or thiopent* or benzodiazepine* or clonazepam* or klonopin or diazepam* or valium* or lorazepam* or ativan* or alprazolam* or xanax* or chlordiazepoxide* or librium* or temazepam* or restoril* or triazolam* or halcion* or midazolam* or nitrazepam* or "sleeping pill*" or zopiclone* or zolpidem* or methaqualone* or quaalude* or mandrax* or cocaine* or crack* or stimulant* or psychostimulant* or amphetamine* or methamphetamine* or methcathinone* or "bath salt*" or cathinone* or inhalant* or mdma or mda or ecstasy or GHB or gamma-hydroxybutyrate* or dissociative* or ketamine* or PCP or polysubstance* or polydrug*)).m_titl.

23 (disorder* adj1 (substance* or drug* or alcohol* or drink* or hallucinogen* or cannabi* or marijuana* or LSD or mescaline* or psilocybin* or hash* or tetrahydrocannabinol* or THC or heroin* or opioid* or opiate* or opium* or codeine* or Dolophine* or methadose* or fentanyl* or actiq* or duragesic* or sublimaze* or morphine* or duramorph* or kadian* or avinza* or hydrocodone* or vicodin* or oxycodone* or oxycontin* or percocet* or percodan* or oxymorphone* or opana* or sedative* or

hypnotic* or anxiolytic* or barbiturate* or amobarbital* or amytal* or pentobarbit* or nembutal* or phenobarbit* or luminal* or secobarbital* or seconal* or tuinal* or Benzylbutylbarbiturate* or Butalbital* or butisol* or thiopent* or benzodiazepine* or clonazepam* or klonopin or diazepam* or valium* or lorazepam* or ativan* or alprazolam* or xanax* or chlordiazepoxide* or librium* or temazepam* or restoril* or triazolam* or halcion* or midazolam* or nitrazepam* or "sleeping pill*" or zopiclone* or zolpidem* or methaqualone* or quaalude* or mandrax* or cocaine* or crack* or stimulant* or psychostimulant* or amphetamine* or methamphetamine* or methcathinone* or "bath salt*" or cathinone* or inhalant* or mdma or mda or ecstasy or GHB or gamma-hydroxybutyrate* or dissociative* or ketamine* or PCP or polysubstance* or polydrug*)).m_titl.

24 18 or 19 or 20 or 21 or 22 or 23

25 17 or 24

26 5 and 13 and 25

27 limit 26 to (human and (180 school age <age 6 to 12 yrs> or 200 adolescence <age 13 to 17 yrs> or "300 adulthood <age 18 yrs and older>" or 320 young adulthood <age 18 to 29 yrs> or 340 thirties <age 30 to 39 yrs> or 360 middle age <age 40 to 64 yrs> or "380 aged <age 65 yrs and older>" or "390 very old <age 85 yrs and older>") and yr="2014 -Current")

28 ("prevention program*" or "prevention intervention*" or "preventing smoking" or "prevent gaming addiction" or "preventive intervention*" or "alcohol prevention" or "abuse prevention" or "prevention of substance*" or "prevention of alcohol" or "prevention of teen*" or "prevention of adolescen*" or "prevention of problem*" or "prevention of disorder*" or "prevention of hazard*" or "prevent* smoking" or "prevent* tobacco" or "substance use prevention" or "tobacco prevention" or "substance prevention" or "universal").m_titl.

29 (secondhand or second-hand or "smoke exposure" or "exposure to tobacco" or "passive smoking" or "passive smoke").m_titl.

30 ((feasibility adj3 study) or (feasibility adj3 trial)).m_titl.

31 **dissociative identity disorder.m_titl.**

32 ("cost-effective*" or "budgetery" or "funding" or "economic evaluation*" or "economic impact*" or "health economic*").m_titl.

33 "laboratory*".m_titl.

34 "policy intervention*".m_titl.

35 "non-treatment seeking".ti,ab.

36 28 or 29 or 30 or 31 or 32 or 33 or 34 or 35

37 27 not 36

Embase and Medline

1 therapy/

2 intervention study/

3 program evaluation/ or program effectiveness/ or program efficacy/ or program impact/

4 treatment outcome/

5 ((treatment* or treated* or posttreat* or post-treat* intervention* or intervene* or therap* or psychotherap* or program*3 or pharmacotherap* or medicat*) adj6 (Patient-reported outcome* or self-report* or patient-

centre* or patient-center* or PRO or PROs or PROM or PROMs or outcome* or efficac* or effect* or success* or impact* or evaluat* or assess*)).ti,ab.

6 1 or 2 or 3 or 4 or 5

7 clinical trial/

8 (randomi?ed or randomly or allocated or assigned or placebo).ab.

9 (RCT or controlled trial* or control trial* or clinical trial* or clinicaltrial* or open label trial* or open trial* or randomi?ed trial* or single-blind trial* or double-blind trial* or triple-blind trial* or blind trial*).ti,ab.

10 7 or 8 or 9

11 qualitative research/

12 (case series or ((naturalistic or case-control) adj1 (stud* or design* or trial*)) or (qualitative adj1 (stud* or research or design* or interview*))).ti,ab.

13 11 or 12

14 10 or 13

15 pathological gambling/

16 (gambl* adj3 (dependen* or disorder* or withdraw* or abus* or addict* or harm* or problem* or pathological or compulsive or misus* or hazardous or high-risk or risky)).m_titl.

17 game addiction/

18 ((game* or gaming*) adj3 (dependen* or disorder* or withdraw* or abus* or addict* or harm* or problem* or pathological or compulsive or misus* or hazardous or high-risk or risky)).m_titl.

19 (addictive behavio?r* or behavio?ral addict*).m_titl.

20 15 or 16 or 17 or 18 or 19

21 (smoking or smoker or smokers or tobacco or nicotine or cigarette*).m_titl.

22 ("e-cigarette*" or ecigarette* or vaping*).m_titl.

23 ("alcoholics" or "alcoholic patient*" or "alcoholism").m_titl.

24 ((injur* or abus* or harm* or problem* or pathological or compulsive or misus* or hazardous) adj2 (substance* or drug* or alcohol* or drink* or hallucinogen* or cannabi* or marijuana* or LSD or mescaline* or psilocybin* or hash* or tetrahydrocannabinol* or THC or heroin* or opioid* or opiate* or opium* or codeine* or Dolophine* or methadose* or fentanyl* or actiq* or duragesic* or sublimaze* or morphine* or duramorph* or kadian* or avinza* or hydrocodone* or vicodin* or oxycodone* or oxycontin* or percocet* or percodan* or oxymorphone* or opana* or sedative* or hypnotic* or anxiolytic* or barbiturate* or amobarbital* or amytal* or pentobarbit* or nembutal* or phenobarbit* or luminal* or secobarbital* or seconal* or tuinal* or Benzylbutylbarbiturate* or Butalbital* or butisol* or thiopent* or benzodiazepine* or clonazepam* or klonopin or diazepam* or valium* or lorazepam* or ativan* or alprazolam* or xanax* or chlordiazepoxide* or librium* or temazepam* or restoril* or triazolam* or halcion* or midazolam* or nitrazepam* or "sleeping pill*" or zopiclone* or zolpidem* or methaqualone* or quaalude* or mandrax* or cocaine* or crack* or stimulant* or psychostimulant* or amphetamine* or methamphetamine* or methcathinone* or "bath salt*" or cathinone* or inhalant* or mdma or mda or ecstasy or GHB or gamma-hydroxybutyrate* or dissociative* or ketamine* or PCP or polysubstance* or polydrug*)).m_titl.

25 ((dependen* or withdraw* or addict*) adj3 (substance* or drug* or alcohol* or drink* or hallucinogen* or cannabi* or marijuana* or LSD or mescaline* or psilocybin* or hash* or tetrahydrocannabinol* or THC or

heroin* or opioid* or opiate* or opium* or codeine* or Dolophine* or methadose* or fentanyl* or actiq* or duragesic* or sublimaze* or morphine* or duramorph* or kadian* or avinza* or hydrocodone* or vicodin* or oxycodone* or oxycontin* or percocet* or percodan* or oxymorphone* or opana* or sedative* or hypnotic* or anxiolytic* or barbiturate* or amobarbital* or amytal* or pentobarbit* or nembutal* or phenobarbit* or luminal* or secobarbital* or seconal* or tuinal* or Benzylbutylbarbiturate* or Butalbital* or butisol* or thiopent* or benzodiazepine* or clonazepam* or klonopin or diazepam* or valium* or lorazepam* or ativan* or alprazolam* or xanax* or chlordiazepoxide* or librium* or temazepam* or restoril* or triazolam* or halcion* or midazolam* or nitrazepam* or "sleeping pill*" or zopiclone* or zolpidem* or methaqualone* or quaalude* or mandrax* or cocaine* or crack* or stimulant* or psychostimulant* or amphetamine* or methamphetamine* or methcathinone* or "bath salt*" or cathinone* or inhalant* or mdma or mda or ecstasy or GHB or gamma-hydroxybutyrate* or dissociative* or ketamine* or PCP or polysubstance* or polydrug*)).m_titl.

26 (disorder* adj1 (substance* or drug* or alcohol* or drink* or hallucinogen* or cannabi* or marijuana* or LSD or mescaline* or psilocybin* or hash* or tetrahydrocannabinol* or THC or heroin* or opioid* or opiate* or opium* or codeine* or Dolophine* or methadose* or fentanyl* or actiq* or duragesic* or sublimaze* or morphine* or duramorph* or kadian* or avinza* or hydrocodone* or vicodin* or oxycodone* or oxycontin* or percocet* or percodan* or oxymorphone* or opana* or sedative* or hypnotic* or anxiolytic* or barbiturate* or amobarbital* or amytal* or pentobarbit* or nembutal* or phenobarbit* or luminal* or secobarbital* or seconal* or tuinal* or Benzylbutylbarbiturate* or Butalbital* or butisol* or thiopent* or benzodiazepine* or clonazepam* or klonopin or diazepam* or valium* or lorazepam* or ativan* or alprazolam* or xanax* or chlordiazepoxide* or librium* or temazepam* or restoril* or triazolam* or halcion* or midazolam* or nitrazepam* or "sleeping pill*" or zopiclone* or zolpidem* or methaqualone* or quaalude* or mandrax* or cocaine* or crack* or stimulant* or psychostimulant* or amphetamine* or methamphetamine* or methcathinone* or "bath salt*" or cathinone* or inhalant* or mdma or mda or ecstasy or GHB or gamma-hydroxybutyrate* or dissociative* or ketamine* or PCP or polysubstance* or polydrug*)).m_titl.

27 21 or 22 or 23 or 24 or 25 or 26

28 20 or 27

29 6 and 14 and 28

30 limit 29 to (human and yr="2014 -Current" and (school child <7 to 12 > or adolescent <13 to 17 > or adult <18 to 64 > or aged <65+ >))

31 ("prevention program*" or "prevention intervention*" or "preventing smoking" or "prevent gaming addiction" or "preventive intervention*" or "alcohol prevention" or "abuse prevention" or "prevention of substance*" or "prevention of alcohol" or "prevention of teen*" or "prevention of adolescen*" or "prevention of problem*" or "prevention of disorder*" or "prevention of hazard*" or "prevent* smoking" or "prevent* tobacco" or "substance use prevention" or "tobacco prevention" or "substance prevention" or "universal" or (never adj4 smoker*) or "prevention trial*").m_titl.

32 (secondhand or second-hand or "smoke exposure" or "exposure to tobacco" or "passive smoking" or "passive smoke").m_titl.

33 ((feasibility adj3 study) or (feasibility adj3 trial) or (feasib* and acceptab*)).m_titl.

34 dissociative identity.m_titl.

35 ("cost-effective*" or "budgetery" or "funding" or "economic evaluation*" or "economic impact*" or "health economic*").m_titl.

36 "laboratory*".m_titl.

37 "policy intervention*".m_titl.

38 "non-treatment seeking".ti,ab.

39 31 or 32 or 33 or 34 or 35 or 36 or 37 or 38

40 30 not 39

41 limit 40 to (embase or medline)

CINAHL

S1 (MH "Program Evaluation+")

S2 (MH "Drug Therapy+") OR (MH "Psychotherapy+")

S3 (MH "Treatment Outcomes+") OR (MH "Patient-Reported Outcomes+")

S4 (TI ((treatment* or treated* or posttreat* or post-treat* intervention* or intervene* or therap* or psychotherap* or program*3 or pharmacotherap* or medicat*) N6 (Patient-reported outcome* or self-report* or patient-centre* or patient-center* or PRO or PROs or PROM or PROMs or outcome* or efficac* or effect* or success* or impact* or evaluat* or assess*))) or (AB ((treatment* or treated* or posttreat* or post-treat* intervention* or intervene* or therap* or psychotherap* or program*3 or pharmaco ...

S5 S1 OR S2 OR S3 OR S4

S6 (MH "Clinical Trials+") OR (MH "Controlled Before-After Studies+") OR (MH "Double-Blind Studies+") OR (MH "Factorial Design+") OR (MH "Nonrandomized Trials+") OR (MH "Pretest-Posttest Design+") OR (MH "Single-Blind Studies+") OR (MH "Solomon Four-Group Design+") OR (MH "Triple-Blind Studies+") OR (MH "Case Control Studies+") OR (MH "Qualitative Studies+")

S7 (AB (randomi#ed or randomly or allocated or assigned or placebo))

S8 (TI (RCT or controlled trial* or control trial* or clinical trial* or clinicaltrial* or open label trial* or open trial* or randomi#ed trial* or single-blind trial* or double-blind trial* or triple-blind trial* or blind trial*)) OR (AB (RCT or controlled trial* or control trial* or clinical trial* or clinicaltrial* or open label trial* or open trial* or randomi#ed trial* or single-blind trial* or double-blind trial* or triple-blind trial* or blind trial*))

S9 (TI (case series or ((naturalistic or case-control) N1 (stud* or design* or trial*)) or (qualitative N1 (stud* or research or design* or interview*))) OR (AB (case series or ((naturalistic or case-control) N1 (stud* or design* or trial*)) or (qualitative N1 (stud* or research or design* or interview*)))

S10 S6 OR S7 OR S8 OR S9

S11 (TI (gambl* N3 (dependen* or disorder* or withdraw* or abus* or addict* or harm* or problem* or pathological or compulsive or misus* or hazardous or high-risk or risky)))

S12 (TI ((game* or gaming*) N3 (dependen* or disorder* or withdraw* or abus* or addict* or harm* or problem* or pathological or compulsive or misus* or hazardous or high-risk or risky)))

S13 (TI (addictive behavio#r* or behavio#ral addict*))

S14	S11 OR S12 OR S13
S15	(TI (smoking or smoker or smokers or tobacco or nicotine or cigarette*))
S16	(TI ("e-cigarette*" or ecigarette* or vaping*))
S17	(TI ("alcoholics" or "alcoholic patient*" or "alcoholism"))
S18	(TI ((injur* or abus* or harm* or problem* or pathological or compulsive or misus* or hazardous) N2 (substance* or drug* or alcohol* or drink* or hallucinogen* or cannabi* or marijuana* or LSD or mescaline* or psilocybin* or hash* or tetrahydrocannabinol* or THC or heroin* or opioid* or opiate* or opium* or codeine* or Dolophine* or methadose* or fentanyl* or actiq* or duragesic* or sublimaze* or morphine* or duramorph* or kadian* or avinza* or hydrocodone* or vicodin* or oxycodone* or oxycontin ...
S19	(TI ((dependen* or withdraw* or addict*) N3 (substance* or drug* or alcohol* or drink* or hallucinogen* or cannabi* or marijuana* or LSD or mescaline* or psilocybin* or hash* or tetrahydrocannabinol* or THC or heroin* or opioid* or opiate* or opium* or codeine* or Dolophine* or methadose* or fentanyl* or actiq* or duragesic* or sublimaze* or morphine* or duramorph* or kadian* or avinza* or hydrocodone* or vicodin* or oxycodone* or oxycontin* or percocet* or percodan* or oxymorphone* or opana* or ...
S20	(TI (disorder* N1 (substance* or drug* or alcohol* or drink* or hallucinogen* or cannabi* or marijuana* or LSD or mescaline* or psilocybin* or hash* or tetrahydrocannabinol* or THC or heroin* or opioid* or opiate* or opium* or codeine* or Dolophine* or methadose* or fentanyl* or actiq* or duragesic* or sublimaze* or morphine* or duramorph* or kadian* or avinza* or hydrocodone* or vicodin* or oxycodone* or oxycontin* or percocet* or percodan* or oxymorphone* or opana* or sedative* or hypnotic* or ...
S21	S15 OR S16 OR S17 OR S18 OR S19 OR S20
S22	S14 OR S21
S23	S5 AND S10 AND S22
S24	S5 AND S10 AND S22 [Limiters - Date Published: 20140101-20190131; Human; Age Groups: Child: 6-12 , Adolescent: 13-18 , Adult: 19-44 , Middle Aged: 45-64 , Aged: 65+ , Aged, 80 and over, All Adult]
S25	(TI ("prevention program*" or "prevention intervention*" or "preventing smoking" or "prevent gaming addiction" or "preventive intervention*" or "alcohol prevention" or "abuse prevention" or "prevention of substance*" or "prevention of alcohol" or "prevention of teen*" or "prevention of adolescen*" or "prevention of problem*" or "prevention of disorder*" or "prevention of hazard*" or "prevent* smoking" or "prevent* tobacco" or "substance use prevention" or "tobacco prevention" or "substance preve ...
S26	(TI (secondhand or second-hand or "smoke exposure" or "exposure to tobacco" or "passive smoking" or "passive smoke"))

S27	(TI ((feasibility N3 study) or (feasibility N3 trial) or (feasib* and acceptab*)))
S28	(TI dissociative identity)
S29	(TI ("cost-effective*" or "budgetery" or "funding" or "economic evaluation*" or "economic impact*" or "health economic*"))
S30	(TI laboratory*)
S31	(TI policy intervention*)
S32	(TI "non-treatment seeking") OR (AB "non-treatment seeking")
S33	S25 OR S26 OR S27 OR S28 OR S29 OR S30 OR S31 OR S32
S34	S24 NOT S33
S1	(MH "Program Evaluation+")
S2	(MH "Drug Therapy+") OR (MH "Psychotherapy+")
S3	(MH "Treatment Outcomes+") OR (MH "Patient-Reported Outcomes+")
S4	(TI ((treatment* or treated* or posttreat* or post-treat* intervention* or intervene* or therap* or psychotherap* or program*3 or pharmacotherap* or medicat*) N6 (Patient-reported outcome* or self-report* or patient-centre* or patient-center* or PRO or PROs or PROM or PROMs or outcome* or efficac* or effect* or success* or impact* or evaluat* or assess*))) or (AB ((treatment* or treated* or posttreat* or post-treat* intervention* or intervene* or therap* or psychotherap* or program*3 or pharmaco ...
S5	S1 OR S2 OR S3 OR S4
S6	(MH "Clinical Trials+") OR (MH "Controlled Before-After Studies+") OR (MH "Double-Blind Studies+") OR (MH "Factorial Design+") OR (MH "Nonrandomized Trials+") OR (MH "Pretest-Posttest Design+") OR (MH "Single-Blind Studies+") OR (MH "Solomon Four-Group Design+") OR (MH "Triple-Blind Studies+") OR (MH "Case Control Studies+") OR (MH "Qualitative Studies+")
S7	(AB (randomi#ed or randomly or allocated or assigned or placebo))

PRISMA flowchart

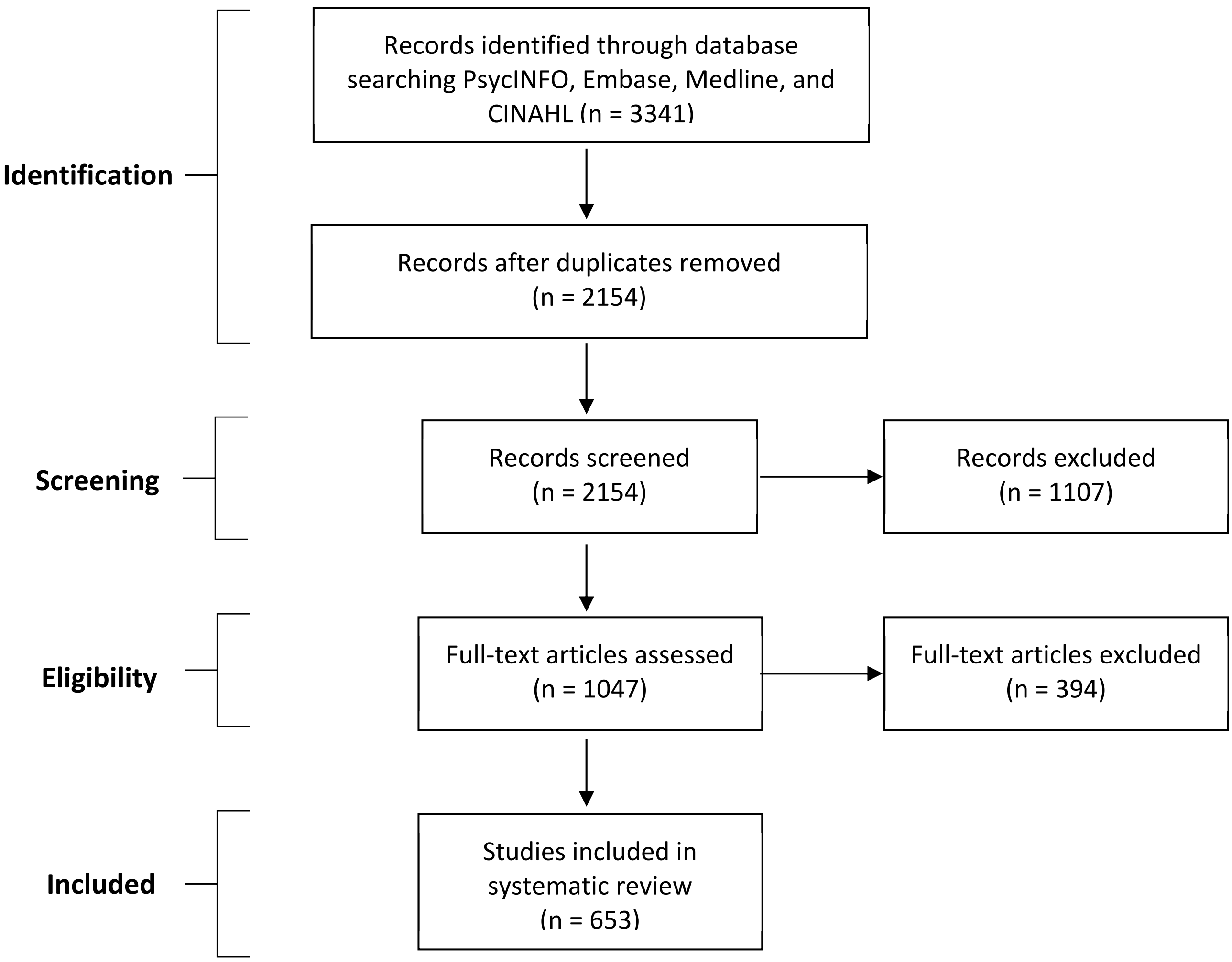

Working Group

Outcome Domains

Overview

All outcomes and case-mix factors were extracted verbatim as described in the studies identified via the systematic review and supplemental sources. The 2088 extracted terms were then synthesised, removing duplicates and collapsing into groupings of meaningfully indistinct terms and assigned a representative label agreed upon by two reviewers (e.g., cognition and cognitive function were collapsed under 'cognitive functioning'). Following inclusion of additional domains suggested by the Working Group and in breakout groups with lived experience representatives and Working Group members, this process resulted in 80 distinct outcome domains, which were considered by the Working Group (listed in next section). Outcome domains considered a priority by lived experience representatives were highlighted to the full Working Group during the process. Working Group members could suggest any additional outcome domains they thought were missing.

Following discussion at the videoconference, in the first round of the Delphi process, the Working Group rated each of the 80 domains on a scale of 1 (should not be included)-9 (essential for inclusion). For ease of Working Group review, the 80 outcome domains were grouped thematically under (1) Symptom Burden and Diagnostic Status, (2) Functioning, (3) Personal Growth and Development, (4) Environment/Eco-System, and (5) Process and Experience. The Working Group members were asked to consider the following factors when assigning their ratings: (1) importance to people with lived experience of the relevant conditions, (2) clinical validity, (3) sensitivity to improvements over care, (4) ease of measurement in routine practice, and (5) cross-cultural validity. Domains that were rated 7-9 by at least 80% of Working Group members were included. Domains that rated 1-3 by at least 80% of Working Group members were excluded. This process was repeated a second time with the undecided domains. In the third and final round, undecided domains were discussed in a further Working Group videoconference, followed by a final binary yes/no vote, with 70% agreement required for inclusion. Comments were shared anonymously after every round to facilitate further discussion and consensus. Support for each outcome domain for each of these rounds is shown in Supplementary S2 and S3.

Supplementary S2. *All Outcome Domains with Consensus Voting and Action for Inclusion in Next Consensus Voting Round*

	Recommend %	80% Consensus Discommend?	80% Consensus Recommend?	50% Majority	60%? Majority	80% of PWLE[1] Panel Recommend?	Result
**Symptom burden and diagnostic status**							
Frequency of activity (Including a quantity/amount element)	**83%**	*No*	**Yes**	-	-	**No**	**Included as Domain**
Health problems specifically associated with disorder	**75%**	*No*	**No**	*Yes*	*Yes*	**Yes**	**Include Next Vote**
Craving (severity and frequency)	**71%**	*No*	**No**	*Yes*	*Yes*	**No**	**Consensus - Exclude**
Symptoms of anxiety	**71%**	No	**No**	Yes	Yes	**Yes**	**Include Next Vote**
Symptoms of depression	**67%**	No	**No**	Yes	Yes	**Yes**	**Include Next Vote**
Harm reduction (or safer use)	**63%**	*No*	**No**	*Yes*	*Yes*	**Yes**	**Include Next Vote**
Symptoms of withdrawal	**63%**	*No*	**No**	*Yes*	*Yes*	**Yes**	**Include Next Vote**
Mortality or overall survival	**50%**	*No*	**No**	*No*	*No*	**No**	**Consensus - Exclude**
Suicidality	**50%**	*No*	**No**	*No*	*No*	**No**	**Consensus - Exclude**
Abstinence	**46%**	*No*	**No**	*No*	*No*	**No**	**Consensus - Exclude**
Risk reduction (pattern of use)	**46%**	*No*	**No**	*No*	*No*	**No**	**Consensus - Exclude**
Symptoms of PTSD	**46%**	No	**No**	No	No	**No**	**Consensus - Exclude**
Overall success of treatment	**38%**	No	**No**	No	No	**No**	**Consensus - Exclude**
Recovery	**33%**	*No*	**No**	*No*	*No*	**No**	**Consensus - Exclude**
Self-harm	**33%**	*No*	**No**	*No*	*No*	**No**	**Consensus - Exclude**

Symptoms of other mental health disorders	**33%**	No	**No**	No	No	**No**	**Consensus - Exclude**
Healthcare service utilisation	**29%**	No	**No**	No	No	**No**	**Consensus - Exclude**
Specialist treatment utilisation	**29%**	No	**No**	No	No	**No**	**Consensus - Exclude**
Subjective effects	**25%**	*No*	**No**	*No*	*No*	**No**	**Consensus - Exclude**
Expenditure related to disorder	**25%**	*No*	**No**	*No*	*No*	**No**	**Consensus - Exclude**
Side effects of medication	**25%**	No	**No**	No	No	**No**	**Consensus - Exclude**
Quit attempts	**17%**	*No*	**No**	*No*	*No*	**No**	**Consensus - Exclude**
Pain	**13%**	*No*	**No**	*No*	*No*	**No**	**Consensus - Exclude**
**Functioning**							
Health-related quality of life	**88%**	No	**Yes**	-	-	Yes	**Included as Domain**
Global functioning	**88%**	No	**Yes**	-	-	Yes	**Included as Domain**
Risk behaviour	**67%**	No	No	Yes	Yes	No	**Consensus - Exclude**
Emotional and behavioural regulation	**58%**	No	No	Yes	No	Yes	**Include Next Vote**
Employment	**58%**	No	No	Yes	No	No	**Include Next Vote**
Vocation/Educational functioning	**58%**	No	No	Yes	No	No	**Include Next Vote**
Recreation and leisure activities	**46%**	No	No	No	No	No	**Consensus - Exclude**
Negative consequences	**42%**	No	No	No	No	No	**Consensus - Exclude**
Interference of treatment with daily life	**42%**	No	No	No	No	No	**Consensus - Exclude**
Engagement with the criminal justice system	**38%**	No	No	No	No	Yes	**Include Next Vote**
Cognitive functioning	**29%**	No	No	No	No	No	**Consensus - Exclude**

Sleep functioning	**29%**	No	No	No	No	No	**Consensus - Exclude**
Physical activity	**25%**	No	No	No	No	No	**Consensus - Exclude**
Impulsivity	**21%**	No	No	No	No	No	**Consensus - Exclude**
Anti-social behaviour	**21%**	No	No	No	No	No	**Consensus - Exclude**
Cognitive bias	**13%**	No	No	No	No	No	**Consensus - Exclude**
Sexual functioning	**4%**	No	No	No	No	No	**Consensus - Exclude**
**Personal Growth and Development**							
Coping skills	**67%**	No	No	Yes	Yes	Yes	**Include Next Vote**
Achievement of goals	**58%**	No	No	Yes	No	No	**Consensus - Exclude**
Self-efficacy	**54%**	No	No	Yes	No	No	**Consensus - Exclude**
Life satisfaction	**50%**	No	No	No	No	Yes	**Include Next Vote**
Health literacy	**46%**	No	No	No	No	No	**Consensus - Exclude**
Motivation	**42%**	No	No	No	No	No	**Consensus - Exclude**
Self-esteem	**42%**	No	No	No	No	Yes	**Include Next Vote**
Autonomy	**38%**	No	No	No	No	No	**Consensus - Exclude**
Readiness to change	**38%**	No	No	No	No	No	**Consensus - Exclude**
Meaning and purpose	**38%**	No	No	No	No	No	**Consensus - Exclude**
Confidence	**33%**	No	No	No	No	No	**Consensus - Exclude**
Acceptance of self	**33%**	No	No	No	No	Yes	**Include Next Vote**
Self-concept/identity	**29%**	No	No	No	No	No	**Consensus - Exclude**
Resilience	**25%**	No	No	No	No	No	**Consensus - Exclude**
Reduction in boredom	**25%**	No	No	No	No	No	**Consensus - Exclude**
Mindfulness	**21%**	No	No	No	No	No	**Consensus - Exclude**
Spirituality	**21%**	No	No	No	No	No	**Consensus - Exclude**
Loneliness	**21%**	No	No	No	No	No	**Consensus - Exclude**
**Environment/Eco-System**							
Relationships	**79%**	No	No	Yes	Yes	Yes	**Include Next Vote**
Social support	**71%**	No	No	Yes	Yes	Yes	**Include Next Vote**

Family relationships	**63%**	No	No	Yes	Yes	Yes	**Include Next Vote**
Sense of belonging or community	**63%**	No	No	Yes	Yes	Yes	**Include Next Vote**
Peer relationships	**58%**	No	No	Yes	No	No	**Consensus - Exclude**
Accommodation status	**42%**	No	No	No	No	Yes	**Include Next Vote**
Perceived stigma	**38%**	No	No	No	No	Yes	**Include Next Vote**
Family functioning	**33%**	No	No	No	No	No	**Consensus - Exclude**
Intimate partner violence	**29%**	No	No	No	No	No	**Consensus - Exclude**
Trauma and negative life events	**29%**	No	No	No	No	No	**Consensus - Exclude**
Financial situation	**29%**	No	No	No	No	No	**Consensus - Exclude**
Family role and responsibilities	**25%**	No	No	No	No	No	**Consensus - Exclude**
Cultural re-integration	**25%**	No	No	No	No	Yes	**Consensus - Exclude**
Financial dependency	**17%**	No	No	No	No	No	**Consensus - Exclude**
Vulnerability	**13%**	No	No	No	No	No	**Consensus - Exclude**
Child welfare and development	**13%**	No	No	No	No	No	**Consensus - Exclude**

[1]PWLE = People With Lived Experience; 24 working group members responded to each domain with a score from '1 = Not Recommended' to '9 = Essential to Have'. For consensus of recommendation, 80% (n=19) of the working group was required to score '7' to '9' to recommend an outcome domain as essential.

Supplementary S3. *Second round of recommendation consensus voting for Outcome Domains that were recommended as essential by 50-79% of the working group in round one.*

**Outcome Domains: Voting for Outcome Domains that received 50-79% in round one (see Supplementary S2)**				
			**General**	
**General Consensus**	***Consensus (%)***	**Consensus 50%**	**Consensus 60%**	**Action**
Do you agree to exclude all other inconclusive domains? (i.e. <50% in first vote)	**87%**	**Yes**	**Yes**	**Consensus - Exclude**
**Do you think any of the below outcome domains should be included in the Set FOR ALL in scope?**				
**Domain**	***Consensus (%)***	**Consensus 50%**	**Consensus 60%**	**Action**
Social support	*65%*	**Yes**	**Yes**	**Consensus - Exclude**
Craving (Severity and Frequency)	*61%*	**Yes**	**Yes**	**Consensus - Exclude**
Health problems specifically associated with disorder	*57%*	**Yes**	**No**	**Consensus - Exclude**
Harm reduction	*48%*	**No**	**No**	**Consensus - Exclude**
Symptoms of withdrawal	*43%*	**No**	**No**	**Consensus - Exclude**
Coping skills	*39%*	**No**	**No**	**Consensus - Exclude**
Life satisfaction	*39%*	**No**	**No**	**Consensus - Exclude**
Employment	*39%*	**No**	**No**	**Consensus - Exclude**
Recovery	*30%*	**No**	**No**	**Consensus - Exclude**
Vocation/Education	*30%*	**No**	**No**	**Consensus - Exclude**
Symptoms of depression	*26%*	**No**	**No**	**Consensus - Exclude**
Pain	*26%*	**No**	**No**	**Consensus - Exclude**
Symptoms of anxiety	*22%*	**No**	**No**	**Consensus - Exclude**
Acceptance of self	*22%*	**No**	**No**	**Consensus - Exclude**
Emotional and behavioural regulation	*22%*	**No**	**No**	**Consensus - Exclude**
Self-esteem	*17%*	**No**	**No**	**Consensus - Exclude**
Engagement with the criminal justice system	*9%*	**No**	**No**	**Consensus - Exclude**
**Do you think any of the below outcome domains should be included in the Set for a SPECIFIC SUB-GROUP?**				
**Outcome Domain**	***Consensus (%)***	**Consensus 50%**	**Consensus 60%**	**Action**
Risk behaviour (illicit drug use)	*61%*	**Yes**	**Yes**	**Consensus - Exclude**
Symptoms of withdrawal (intervention specific)	*57%*	**Yes**	**No**	**Consensus - Exclude**

Expenditure related to disorder (Gambling)	*52%*	**Yes**	**No**	**Consensus - Exclude**
Pain (opioids)	*35%*	**No**	**No**	**Consensus - Exclude**
Side effects of medication (intervention specific)	*35%*	**No**	**No**	**Consensus - Exclude**
Overall survival (opioids)	*26%*	**No**	**No**	**Consensus - Exclude**
Engagement with the criminal justice system	*26%*	**No**	**No**	**Consensus - Exclude**

24 working group members responded to each domain with a score from '1 = Not Recommended' to '9 = Essential to Have'. For consensus of recommendation 70% (n=17) of the working group was required to score '7' to '9' to recommend an outcome domain as essential.

List of 80 unique outcome domains considered

Outcome domains selected for inclusion in the Set are presented in bold.

Symptoms and diagnoses

1. Abstinence
2. Craving
3. Expenditure related to disorder
4. Harm reduction (or safer use)
5. Health problems specifically associated with disorder
6. Healthcare service utilisation
7. Mortality or overall survival
8. Overall success of treatment
9. Pain
10. **Quantity x frequency of behaviour/substance use**
11. Quit attempts
12. Recovery (capital)
13. Risk reduction (pattern of use)
14. Self-harm
15. **Severity of dependence/Symptom burden**
16. Side effects of medication
17. Specialist treatment utilisation
18. Subjective effects
19. Suicidality (ideation, planning and intent)
20. Symptoms of anxiety
21. Symptoms of depression
22. Symptoms of other mental health disorders
23. Symptoms of PTSD
24. Symptoms of withdrawal

Functioning

25. Cognitive bias
26. Cognitive functioning
27. Emotional and behavioural regulation
28. Employment
29. Engagement with the criminal justice system
30. **Global functioning**
31. **Health-related quality of life**
32. Impulsivity
33. Interference of treatment with daily life
34. Negative consequences
35. **Overall mental health and well-being**
36. **Overall physical health and well-being**
37. Physical activity
38. **Psychosocial functioning**
39. Recreation and leisure activities
40. Risk behaviour or harms
41. Sexual functioning
42. Sleep functioning
43. Vocation/Education

Personal growth and development

44. Acceptance of self
45. Achievement of goals
46. Autonomy
47. Confidence
48. Coping skills
49. Health literacy
50. Life satisfaction
51. Loneliness
52. Meaning and purpose
53. Mindfulness
54. Motivation
55. Readiness to change
56. Reduction in boredom
57. Resilience
58. Self-concept/Identity
59. Self-efficacy
60. Self-esteem
61. Spirituality

Environment

62. Accommodation status
63. Child welfare and development
64. Cultural re-integration
65. Family functioning
66. Family relationships
67. Family role and responsibilities
68. Financial dependency
69. Financial situation
70. Intimate partner violence
71. Peer relationships
72. Perceived stigma
73. (Intimate) relationships
74. Sense of belonging or community
75. Social support
76. Trauma and negative life events
77. Vulnerabilities

Process/Experience

78. Therapeutic alliance
79. Treatment adherence, compliance and engagement
80. Treatment experience and satisfaction

Outcome Measures

Overview

The pool of outcome measures consisted of 387 measures in total, of which 158 were mapped to at least one of the outcome domains voted for inclusion in the Set (listed in next section). Outcome measures were mapped to outcome domains by reviewing the the original development papers and also looking at any subscales. In order to identify the most relevant measures and make the best use of the Working Group's time during the videoconference, reduction of this pool of measures followed three steps before presentation at the full Working Group videoconference: initial feasibility assessment, detailed psychometric evaluation, and shortlisting discussion with interested Working Group members. Full details of all three stages of this process were shared with the full Working Group, with the opportunity for Working Group members to flag anything for further discussion.

To pass the feasibility assessment, a measure had to meet four criteria: comprehensive domain coverage, free to use, some validation completed, and takes less than 20 minutes to complete. These criteria were discussed with, and endorsed by, the Working Group. Measures that passed the feasibility assessment underwent full psychometric evaluation in line with International Society for Quality of Life Research (ISOQOL) guidelines,[1] including examination of validity (content, construct, sensitivity to change), reliability (test-retest, internal consistency), ease of interpretation, burden, translations available, and coverage of scope and age groups. To collate the psychometric information, relevant existing studies were reviewed in the following order until all psychometric criteria were appraised: the original validation study, studies identified through the ePROVIDE set of databases of patient-reported outcome measures,[2] systematic reviews, primary studies ordered by recency and presence of measure name in title, and finally studies identified via targeted searching of the measure name with key psychometric terms (to fill any gaps in information). The psychometric information was then summarised and considered in detail by interested Working Group members at breakout shortlisting discussions. Shortlisted measures were considered in full by the full Working Group, supported by summaries of each measure's performance on the outlined ISOQOL categories.

We include below PowerPoint slides illustrating how the summarised information was presented to the Working Group on the calls to facilitate discussion and decision making. In this example, the project team presented the PGSI as the proposed measure for gambling symptoms, based on consideration of the outlined criteria. In other scenarios, multiple options were presented for consideration, facilitating group discussion on the pros and cons of each option.

Severity of Dependence: Gambling*

Gambling options discussed in detail

CRITERION			CPGI	PGSI	VGS	NODS-17
Clinical pop?			?			
VALIDITY	Sensitivity to change					
	Content validity	DSM-5	5 of 9	4 of 9	8 of 9	9 of 9
		ICD-11	2-3 of 3	2-3 of 3	3 of 3	3 of 3
	Construct validity					
RELIABILITY	Test-retest					
	Internal consistency					
TRANSLATION	> 1 language versions		2+	7+	2+	3+
BURDEN	Burden (Items)		31-33	9	21	17
	Scoring					
TOOL ESTABLISHED	Citations		1400	1400	46	795
	Year		2001	2001	2004	1999
REPORTERS	Age coverage		18+	13-87	12-67	18-78
	Reporter		Self	Self	Self	Self
	Recall		12 months	12 months	12 months	12 months

**Note: Gaming will be discussed at the next call*

Severity of Dependence: Gambling

Proposal: PGSI
Rationale:

- ✔ Shortest
- ✔ Strongest psychometrics
- ✔ Most well-established
- ✔ Most languages
- ✔ Validated in adolescents and adults

- x Covers fewest DSM criteria: 4 of 9

Thinking about the last 12 months…

Have you bet more than you could really afford to lose?
0 Never. **1** Sometimes. **2** Most of the time. **3** Almost always.

Still thinking about the last 12 months, have you needed to gamble with larger amounts of money to get the same feeling of excitement?
0 Never. **1** Sometimes. **2** Most of the time. **3** Almost always.

When you gambled, did you go back another day to try to win back the money you lost?
0 Never. **1** Sometimes. **2** Most of the time. **3** Almost always.

Have you borrowed money or sold anything to get money to gamble?
0 Never. **1** Sometimes. **2** Most of the time. **3** Almost always.

Have you felt that you might have a problem with gambling?
0 Never. **1** Sometimes. **2** Most of the time. **3** Almost always.

Has gambling caused you any health problems, including stress or anxiety?
0 Never. **1** Sometimes. **2** Most of the time. **3** Almost always.

Have people criticized your betting or told you that you had a gambling problem, regardless of whether or not you thought it was true?
0 Never. **1** Sometimes. **2** Most of the time. **3** Almost always.

Has your gambling caused any financial problems for you or your household?
0 Never. **1** Sometimes. **2** Most of the time. **3** Almost always.

Have you felt guilty about the way you gamble or what happens when you gamble?
0 Never. **1** Sometimes. **2** Most of the time. **3** Almost always.

Severity of Dependence: Gambling

Proposal: PGSI
DSM coverage:

- ✔ Gambling with increasing amounts of money
- ✔ Wanting to cut down/stop but not managing to
- ✔ Chasing losses
- ✔ Relying on others for financial support due to gambling

- x Spending a lot of time thinking/reliving/planning gambling
- x Jeopardising significant relationship, job, or education/career opportunity due to gambling
- x Withdrawal: restless/irritable when trying to cut down
- x Gambles when feeling distressed
- x Lying to conceal gambling

Copyright © 2018 by the International Consortium for Health Outcomes Measurement. All rights reserved.

Severity of Dependence: Gambling

1. **Do you support the PGSI?**
   - Why/why not?
2. **Is the relatively lower coverage of DSM criteria an issue (cf. VGS and NODS)?**
3. **Is the 12-month recall period an issue?**
   - Could we reduce it if needed?

List of initial 158 measures mapped to one or more included outcome domain; more measures included during process

1. Addiction Severity Index (ASI)
2. Adolescent Diagnostic Interview (ADI)
3. Adolescent Drug Abuse Diagnosis (ADAD)
4. Alcohol Dependence Data Questionnaire (SADD)
5. Alcohol Dependence Quality of Life (ALQoL 9)
6. Alcohol Dependence Scale (ADS)
7. Alcohol Involvement Scale (AIS)
8. Alcohol Use Disorders Identification Test (AUDIT)
9. Alcohol Use Questionnaire (AUQ)
10. Alcohol, Smoking, and Substance Involvement Screening Test (ASSIST)
11. Australian Treatment Outcome Profile (ATOP)
12. Behavior and Symptom Identification Scale (BASIS)
13. Brief Addiction Monitor (BAM)
14. Brief Psychiatric Rating Scale (BPRS)
15. Brief Symptom Inventory (BSI)
16. CAGE Questionnaire
17. California Psychological Inventory (CPI)
18. Canadian Problem Gambling Index (CPGI)
19. Cannabis Problems Questionnaire (CPQ)
20. Child Behavior Checklist (CBCL)
21. Children's Global Assessment Scale (CGAS)
22. Christo Inventory for Gambling Services (CISS)
23. Christo Inventory for Substance-misuse Services (CISS)
24. Christo Inventory of Drugs (CID)
25. Cigarette Dependence Scale (CDS)
26. Clinical Global Impression (CGI) Scale
27. Clinical Outcomes in Routine Evaluation Outcome Measure (CORE-OM)
28. College Alcohol Problems Scale (CAPS)
29. Community Reinforcement Approach Happiness Scale (CRA-HS)
30. Composite International Diagnostic Interview (CIDI)
31. CRAFFT
32. Criteria for Control of Pathological Gambling Questionnaire (CCPGQ)
33. CUGE Questionnaire
34. Customary Drinking and Drug Use Record (Cddr)
35. Daily Drinking Questionnaire
36. Drug Abuse Screening Test (DAST)
37. Drug History Questionnaire (DHQ)
38. Drug Use Disorder Identification Test (DUDIT)
39. DSM-III-R Checklist
40. EuroQol Five-dimensional (EQ-5D) Descriptive System
41. Fagerstrom Test for Nicotine Dependence (FTND)
42. Fagerstrom Tolerance Questionnaire (FTQ)
43. Five-Shot Questionnaire
44. Form 90
45. GAIN Instruments
46. Gambling Activities Screen (Gas)
47. Gambling Activity Record
48. Gambling Follow-Up Scale (GFS)
49. Gambling Symptom Assessment Scale (GSAS)
50. Gambling Treatment Outcome Monitoring System (GAMTOMS)
51. Gambling-Related Questions
52. General Health Questionnaire (GHQ)
53. General Self Efficacy Scale (GSES)
54. Global Assessment of Functioning (GAF) Scale
55. Global Assessment of Individual Needs Short Screener (GAINSS)
56. Global Assessment of Recent Stress (GARS)
57. Global Clinical Outcome
58. Health Questionnaire (HQ)
59. Heaviness of Smoking Index (Hsi)
60. Hooked On Nicotine Checklist
61. Hopkins Symptom Checklist
62. ICD-10 Symptom Checklist for Mental Disorders
63. ICEpop CAPability Instrument for Adults (ICECAP-A)
64. Impact of Event Scale (IES)
65. Impaired Control Scale (ICS)
66. Inventory of [Gambling/Drug Taking] Situations
67. Inventory of Depression and Anxiety Symptoms (Idas)
68. Inventory of Drinking Situations (IDS)
69. Inventory of Drug Use Consequences (Induc)
70. Inventory of Drug-Taking Situations (Idts)
71. Inventory of Interpersonal Problems
72. Kessler Psychological Distress Scale
73. Lancashire Quality of Life Profile (LQoL)
74. Leeds Dependence Questionnaire (Ldq)
75. Lie-Bet Questionnaire (L/BQ)
76. Maudsley Addiction Profile (MAP)
77. Measurements in Addiction for Triage and Evaluation (MATE)
78. Michigan Alcoholism Screening Test (MAST)
79. Mini International Neuropsychiatric Interview (MINI)
80. Monitoring Area and Phase System - Intake (MAPS - Intake)
81. Monitoring Area and Phase System - Out (discharge) (MAPS - Out)
82. Monitoring Area and Phase System - Up (MAPS-Up)
83. Munich-Composite International Diagnostic Interview (M-CIDI)
84. National Opinion Research Center Screen for Gambling Problems (NORC DSM-IV/NODS)
85. Nicotine Dependence Syndrome Scale (Ndss)
86. NIH - General life satisfaction
87. NIH - Perceived Stress
88. NIH - Positive affect
89. NIH - Specific Life Satisfaction
90. NIH Perceived Stress
91. NIMH Diagnostic Interview Schedule IV
92. NIMH Self-Rating Scale (NIMH-SR)
93. Opiate Treatment Index (OTI)
94. Outcome Rating Scale (Ors)
95. Pathological Gambling Behavioural Scale (PGBS)
96. Pathological Gambling Diagnostic Interview (PGDI)
97. Perceived Competence Scale for Children (PCSC)
98. Perceived Stress Scale (PSS)

99. Personal Experience Inventory for Adults (PEI-A)
100. Personal Experience Screening Questionnaire (PESQ)
101. Problem Gambling Severity Index (Pgsi) (of the Canadian Problem Gambling Index)
102. Problem Oriented Screening Instrument for Teenagers (POSIT)
103. Problematic Online Game Use Scale (Pogus)
104. Profile of Mood States (POMS)
105. PROMIS - General Life Satisfaction
106. PROMIS - Physical Function 10a
107. PROMIS - Profile 29 +
108. PROMIS - Severity of Substance Use (past 30 days)
109. PROMIS - Smoking Nicotine Dependence for All Smokers
110. PROMIS - Smoking Nicotine Dependence for Daily Smokers
111. PROMIS Alcohol Use 7a
112. PROMIS Global Health - Mental
113. PROMIS Global Health - Physical
114. PROMIS Global Health -7a
115. PROMIS Prescription Pain Medication Misuse
116. PROMIS Psychosocial Illness Impact (Negative)
117. PROMIS Psychosocial Illness Impact (Positive)
118. PROMIS -Severity of Substance Use (Past 3 months)
119. PROMIS- Smoking Negative Psychosocial Expectancies for Daily or Non-Daily Smokers
120. Psychological General Well Being Schedule (PGWB)
121. Quality of Life Enjoyment and Satisfaction Questionnaire (QLESQ)
122. Quality of Life in Drug Addiction Questionnaire
123. Quality of Life Inventory (QoLI)
124. Rapid Alcohol Problems Screen (RAPS)
125. Recovery Progression Measure (RPM)
126. Relapse Interview
127. Retrospective Quality of Life Scale
128. Revised Clinical Interview Schedule (CIS-R)
129. Ryff's Scales of Pychological Well-Being (RPWB)
130. Schedule For Clinical Assessment in Neuropsychiatry (Scan)
131. Screening Questionnaire of Common Mental Disorders
132. Self-Report Dysexecutive Questionnaire (Dexs)
133. Semistructured Assessment for the Genetics of Alcoholism (Ssaga)
134. Severity of Alcohol Dependence Scale (SADS) [la escala de la intensidad de la dependencia al alcohol (EIDA)]
135. Severity of Dependence Scale
136. Severity of Opiate Dependence Questionnaire (SODQ)
137. Sheehan Disability Scale (Sds)
138. Short Alcohol Dependence Data Questionnaire (Sadd)
139. Short Form Health Survey (SF)
140. Short Index of Problems (SIP)
141. Smoking Cessation Quality of Life (SCQoL) Questionnaire
142. Sociotropy-Autonomy Scale (Sas)
143. South Oaks Gambling Screen (Sogs)
144. Strengths and Difficulties Questionnaire (SDQ)
145. Structured Clinical Interview For DSM Diagnosis (SCID)
146. Subjective Units of Distress Scale (Suds)
147. Temperament and Character Inventory (TCI)
148. Temporal Experience of Pleasure Scale (Teps)
149. The Health-Related Quality of Life for Drug Abusers Test (HRQOLDA_T)
150. Tidslinjeskema (Danish)
151. Timeline Follow-Back Questionnaire (TFBQ)
152. Tobacco Dependence Screener
153. Tobacco, Alcohol, Prescription Medications, and Substance Use/Misuse (TAPS) Tool
154. Tolerance, Worried, Eye-opener, Amnesia, K/Cutdown (Modified TWEAK)
155. Toronto Alexithymia Scale (Tas20)
156. Victoria Gambling Screen (Vgs)
157. Wisconsin Inventory of Smoking Dependence Motives (Wisdm)
158. World Health Organisation Quality of Life (Whoqol

Supplementary S4. *All included measures, domain and area coverage, recommendation by working group, and primary reasoning for exclusion.*

**Areas**	**Domain/s**	**Measure** *(Measure were suggested by working group members if not present during deliberation)*	**Consensus**	**Reasonings for exclusion (***If no reasoning provided, the measure did not meet the prescribed minimum criteria threshold (e.g. did not meet feasibility assessment, psychometric evaluation, barriers to availability or accessibility)*
A, D, S	SB	Addiction Severity Index (ASI)	Excluded	Consensus recommended alternative measure after consideration
A	SB x FQ[1]	Adolescent Alcohol Involvement Scale (AAIS)	Excluded	Content: covers ≤ 4 DSM criteria
-	SB x FQ	Adolescent Diagnostic Interview (ADI)	Excluded	Excluded for population.
D	SB x FQ	Adolescent Drug Abuse Diagnosis (ADAD)	Excluded	-
A	-	Alcohol Dependence Data Questionnaire (SADD)	Excluded	-
A	GF x QoL[2]	Alcohol Dependence Quality of Life (ALQoL 9)	Excluded	-
A	SB x FQ	Alcohol Dependence Scale (ADS)	Excluded	-
A	SB x FQ	Alcohol Involvement Scale (AIS)	Excluded	-
A	FQ	Alcohol Use Disorders Identification Test (AUDIT)	Excluded	Sensitivity to change - the AUDIT was designed as a screening measure and given the nature of the questions; the group are unsure whether it would be sensitive to change (no validations reported). Recall period - given the purpose of the AUDIT for screening, its recall period is either 1 year, or lifetime. This makes it difficult to use as a regular measure of outcome. The suggestion from the project team was that the recall period would be difficult to change a) given the AUDITs established use worldwide as is, and ii) due to the fact the recall period is 'baked in' to the measure; the time period forming part of the response options as well as the question, meaning substantial change would be required - this would require author permission.
A	SB x FQ	Alcohol Use Questionnaire (AUQ)	Excluded	Brought back in from language exclusion but was not discussed.<br>PT propose that this is excluded as has not been validated in a clinical population.
A	SB x FQ	Alcohol, Smoking, and Substance Involvement Screening Test (ASSIST)	Excluded	Content: covers ≤ 4 DSM criteria
**A, D, S**	**FQ**	**Australian Treatment Outcome Profile (A/TOP)**	**Included**	**-**
-		Behavior and Symptom Identification Scale (BASIS)	Excluded	-
A, D, S	WB[3]; Functioning	Brief Addiction Monitor (BAM)	Excluded	Psychometric Evidence
-	MH[4]	Brief Psychiatric Rating Scale (BPRS)	Excluded	-
-	-	Brief Symptom Inventory (BSI)	Excluded	-
-	-	CAGE Questionnaire	Excluded	-
MH	-	California Psychological Inventory (CPI)	Excluded	-

**Areas**	**Domain/s**	**Measure** *(Measure were suggested by working group members if not present during deliberation)*	**Consensus**	**Reasonings for exclusion (***If no reasoning provided, the measure did not meet the prescribed minimum criteria threshold (e.g. did not meet feasibility assessment, psychometric evaluation, barriers to availability or accessibility)*
Gambling	-	Canadian Problem Gambling Index (CPGI)	Excluded	None of the identified gambling measures had definitive evidence that they were well suited as outcome measures
D		Cannabis Problems Questionnaire (CPQ)	Excluded	-
-	-	Child Behavior Checklist (CBCL)	Excluded	Target age out of scope.
-	-	Children's Global Assessment Scale (CGAS)	Excluded	Target age out of scope.
Gambling	-	Christo Inventory for Gambling Services (CISS)	Excluded	None of the identified gambling measures had definitive evidence that they were well suited as outcome measures
D	-	Christo Inventory for Substance-misuse Services (CISS)	Excluded	-
D	-	Christo Inventory of Drugs (CID)	Excluded	-
S	SB	Cigarette Dependence Scale (CDS)	Excluded	The PROMIS is shorter, was more popular in recommendation voting, and is consistent with the other measures already recommended for the set. For these reasons the PROMIS was recommended over the CDS by consensus (96%).
-	-	Clinical Global Impression (CGI) Scale	Excluded	
-	WB x GF	Clinical Outcomes in Routine Evaluation Outcome Measure (CORE-OM)	Excluded	Length and breadth of coverage
A	-	College Alcohol Problems Scale (CAPS)	Excluded	-
-	-	Community Reinforcement Approach Happiness Scale (CRA-HS)	Excluded	-
-	-	Composite International Diagnostic Interview (CIDI)	Excluded	-
-	-	CRAFFT	Excluded	-
Gambling	-	Criteria for Control of Pathological Gambling Questionnaire (CCPGQ)	Excluded	None of the identified gambling measures had definitive evidence that they were well suited as outcome measures
-	-	CUGE Questionnaire	Excluded	
A, D	SB x FQ	Customary Drinking and Drug Use Record (Cddr)	Excluded	-
-	SB x FQ	Daily Drinking Questionnaire	Excluded	Not validated in a clinical population (primarily in university students)
A, D	SB x FQ	Drug Abuse Screening Test (DAST) [For both Alcohol and drugs]	Excluded	Similar to, but not as robust as DUDIT, and subject to the same issues.
D	SB x FQ	Drug History Questionnaire (DHQ)	Excluded	-
D	FQ	Drug Use Disorder Identification Test (DUDIT)	Excluded	Difficult to summarise psychometrics as different versions have been used; will not be able to benchmark across services very well; very brief per substance, potential for high burden for poly-substance users.
-	-	DSM-III-R Checklist	Excluded	-

Areas	Domain/s	**Measure** *(Measure were suggested by working group members if not present during deliberation)*	Consensus	**Reasonings for exclusion** (*If no reasoning provided, the measure did not meet the prescribed minimum criteria threshold (e.g. did not meet feasibility assessment, psychometric evaluation, barriers to availability or accessibility)*)
Generic	GF x QoL	EuroQol Five-dimensional (EQ-5D) Descriptive System	Excluded	EQ-5D was a standout however the PROMIS Global Health was preferred for measurement of generic functioning (19% vs. 81%).
S	SB x FQ	Fagerstrom Test for Nicotine Dependence (FTND)	Excluded	Poor internal consistency (generally ≤ .7)
-	-	Fagerstrom Tolerance Questionnaire (FTQ)	Excluded	-
-	-	Five-Shot Questionnaire	Excluded	-
-	-	Form 90	Excluded	-
A, D	-	GAIN Instruments	Excluded	Consensus on removal of GAIN due to cost
Gambling	-	Gambling Activities Screen (Gas)	Excluded	None of the identified gambling measures had definitive evidence that they were well suited as outcome measures. The PGSI was proposed as the severity of gambling measure and supported through consensus.
Gambling	-	Gambling Activity Record	Excluded	None of the identified gambling measures had definitive evidence that they were well suited as outcome measures. The PGSI was proposed as the severity of gambling measure and supported through consensus.
Gambling	-	Gambling Follow-Up Scale (GFS)	Excluded	None of the identified gambling measures had definitive evidence that they were well suited as outcome measures. The PGSI was proposed as the severity of gambling measure and supported through consensus.
Gambling	-	Gambling Symptom Assessment Scale (GSAS)	Excluded	None of the identified gambling measures had definitive evidence that they were well suited as outcome measures. The PGSI was proposed as the severity of gambling measure and supported through consensus.
Gambling	-	Gambling Treatment Outcome Monitoring System (GAMTOMS)	Excluded	None of the identified gambling measures had definitive evidence that they were well suited as outcome measures. The PGSI was proposed as the severity of gambling measure and supported through consensus.
Gambling	-	Gambling-Related Questions	Excluded	None of the identified gambling measures had definitive evidence that they were well suited as outcome measures. The PGSI was proposed as the severity of gambling measure and supported through consensus.
Overall	MH x PH x QoL	General Health Questionnaire (GHQ)	Excluded	-
Overall	-	General Self Efficacy Scale (GSES)	Excluded	-
-	GS	Global Assessment of Functioning (GAF) Scale	Excluded	-
-	-	Global Assessment of Individual Needs Short Screener (GAINSS)	Excluded	-
-	-	Global Assessment of Recent Stress (GARS)	Excluded	-
-	-	Global Clinical Outcome	Excluded	-
-	-	Health Questionnaire (HQ)	Excluded	-

Areas	Domain/s	**Measure** *(Measure were suggested by working group members if not present during deliberation)*	Consensus	**Reasonings for exclusion** (*If no reasoning provided, the measure did not meet the prescribed minimum criteria threshold (e.g. did not meet feasibility assessment, psychometric evaluation, barriers to availability or accessibility)*)
**S**	**SB x FQ**	**Heaviness of Smoking Index (HSI)**	**Included**	**One item included: How soon after you wake do you have your first cigarette? (91%). Poor internal consistency (generally ≤ .7) and the brevity creates inequity in coverage compared to other measures**
S	SB x FQ	Hooked On Nicotine Checklist	Excluded	Content: covers ≤ 4 DSM criteria
-	-	Hopkins Symptom Checklist	Excluded	-
Overall	MH	ICD-10 Symptom Checklist for Mental Disorders	Excluded	-
D	WB x GF	ICEpop CAPability Instrument for Adults (ICECAP-A)	Excluded	Discarded for breadth of coverage
-	-	Impact of Event Scale (IES)	Excluded	-
-	SB x FQ	Impaired Control Scale (ICS)	Excluded	Long length and poor psychometrics
Gambling, D	SB x FQ	Inventory of [Gambling/Drug Taking] Situations	Excluded	None of the identified gambling measures had definitive evidence that they were well suited as outcome measures. The PGSI was proposed as the severity of gambling measure and supported through consensus.
Overall	MH x WB	Inventory of Depression and Anxiety Symptoms (Idas)	Excluded	-
-		Inventory of Drinking Situations (IDS)	Excluded	-
-	SB x FQ	Inventory of Drug Use Consequences (Induc)	Excluded	-
-	-	Inventory of Drug-Taking Situations (Idts)	Excluded	-
-	-	Inventory of Interpersonal Problems	Excluded	-
Stress	MH	Kessler Psychological Distress Scale	Excluded	-
-	-	Lancashire Quality of Life Profile (LQoL)	Excluded	-
-	-	Leeds Dependence Questionnaire (Ldq)	Excluded	-
-	-	Lie-Bet Questionnaire (L/BQ)	Excluded	-
A, S, D	SB x FQ	Maudsley Addiction Profile (MAP)	Excluded	TOP favoured over MAP; ability to break down by weeks perceived as more useful and a closer counterpart to the TLFB.
A	SB x FQ	Measurements in Addiction for Triage and Evaluation (MATE)	Excluded	-
A	SB x FQ	Michigan Alcoholism Screening Test (MAST)	Excluded	-
-	-	Mini International Neuropsychiatric Interview (MINI)	Excluded	-
-	-	Monitoring Area and Phase System - Intake (MAPS - Intake)	Excluded	-
--	-	Monitoring Area and Phase System - Out (discharge) (MAPS - Out)	Excluded	-
-	-	Monitoring Area and Phase System - Up (MAPS-Up)	Excluded	-

Areas	Domain/s	**Measure** *(Measure were suggested by working group members if not present during deliberation)*	Consensus	**Reasonings for exclusion (***If no reasoning provided, the measure did not meet the prescribed minimum criteria threshold (e.g. did not meet feasibility assessment, psychometric evaluation, barriers to availability or accessibility)***)**
-	-	Munich-Composite International Diagnostic Interview (M-CIDI)	Excluded	-
S	SB x FQ	National Opinion Research Center Screen for Gambling Problems (NORC DSM-IV/NODS)	Excluded	None of the identified gambling measures had definitive evidence that they were well suited as outcome measures. After consideration of psychometrics the PGSI was recommended.
S	SB	Nicotine Dependence Syndrome Scale (Ndss)	Excluded	Content: covers ≤ 4 DSM criteria
Overall	QoL	NIH - General life satisfaction	Excluded	-
Overall	MH x WB	NIH - Perceived Stress	Excluded	-
Overall	MH x WB	NIH - Positive affect	Excluded	-
Overall	MH x WB	NIH - Specific Life Satisfaction	Excluded	-
Overall	MH x WB	NIH Perceived Stress	Excluded	-
		NIMH Diagnostic Interview Schedule IV	Excluded	-
		NIMH Self-Rating Scale (NIMH-SR)	Excluded	-
D	SB x FQ	Opiate Treatment Index (OTI)	Excluded	-
		Outcome Rating Scale (Ors)	Excluded	-
Gambling	SB x FQ	Pathological Gambling Behavioural Scale (PGBS)	Excluded	None of the identified gambling measures had definitive evidence that they were well suited as outcome measures. The PGSI was proposed as the severity of gambling measure and supported through consensus.
Gambling	SB x FQ	Pathological Gambling Diagnostic Interview (PGDI)	Excluded	None of the identified gambling measures had definitive evidence that they were well suited as outcome measures. The PGSI was proposed as the severity of gambling measure and supported through consensus.
-	-	Perceived Competence Scale for Children (PCSC)	Excluded	-
-	-	Perceived Stress Scale (PSS)	Excluded	-
-	-	Personal Experience Inventory for Adults (PEI-A)	Excluded	-
-	-	Personal Experience Screening Questionnaire (PESQ)	Excluded	-
Gambling	SB	Problem Gambling Severity Index (Pgsi) (of the Canadian Prob	**Included**	None of the identified gambling measures had definitive evidence that they were well suited as outcome measures. The PGSI was proposed as the severity of gambling measure and supported through consensus.
-	-	Problem Oriented Screening Instrument for Teenagers (POSIT)	Excluded	-
Gaming	-	Problematic Online Game Use Scale (Pogus)	Excluded	-
MH	-	Profile of Mood States (POMS)	Excluded	-
Overall	WB	PROMIS - General Life Satisfaction	Excluded	-
Overall	PH x WB	PROMIS - Physical Function 10a	Excluded	-

**Areas**	**Domain/s**	**Measure** *(Measure were suggested by working group members if not present during deliberation)*	**Consensus**	**Reasonings for exclusion (***If no reasoning provided, the measure did not meet the prescribed minimum criteria threshold (e.g. did not meet feasibility assessment, psychometric evaluation, barriers to availability or accessibility)*
		PROMIS - Profile 29 +	Excluded	-
D	SB	PROMIS - Severity of Substance Use (past 30 days)	**Included**	-
S	SB	PROMIS - Smoking Nicotine Dependence for All Smokers 8a	**Included**	-
S	SB	PROMIS - Smoking Nicotine Dependence for Daily Smokers	Excluded	-
A	SB	PROMIS Alcohol Use 7a	**Included**	-
Overall	PH x WB	PROMIS Global Health - Mental (single item)	**Included**	-
Overall	MH x WB	PROMIS Global Health – Physical (single item)	**Included**	-
Overall	MH x PH x WB	PROMIS Global Health -7a	Excluded	-
-	-	PROMIS Prescription Pain Medication Misuse	Excluded	-
-	-	PROMIS Psychosocial Illness Impact (Negative)	Excluded	-
-	-	PROMIS Psychosocial Illness Impact (Positive)	Excluded	-
-	SB x FQ	PROMIS -Severity of Substance Use (Past 3 months)	Excluded	-
S	SB x FQ	PROMIS- Smoking Negative Psychosocial Expectancies for Daily	Excluded	-
-	MH	Psychological General Well Being Schedule (PGWB)	Excluded	-
-	-	Quality of Life Enjoyment and Satisfaction Questionnaire (QL	Excluded	-
D	QoL	Quality of Life in Drug Addiction Questionnaire	Excluded	-
	QoL	Quality of Life Inventory (QoLI)	Excluded	-
A	SB x FQ	Rapid Alcohol Problems Screen (RAPS)	Excluded	-
	-	Recovery Progression Measure (RPM)	Excluded	-
-	-	Relapse Interview	Excluded	-
-	QoL	Retrospective Quality of Life Scale	Excluded	-
-		Revised Clinical Interview Schedule (CIS-R)	Excluded	-
-	MH x WB	Ryff's Scales of Pychological Well-Being (RPWB)	Excluded	-
-		Schedule For Clinical Assessment in Neuropsychiatry (Scan)	Excluded	-
-	MH	Screening Questionnaire of Common Mental Disorders	Excluded	-
-		Self-Report Dysexecutive Questionnaire (Dexs)	Excluded	-
A	SB x FQ	Semistructured Assessment for the Genetics of Alcoholism (Ssaga)	Excluded	-

**Areas**	**Domain/s**	**Measure** *(Measure were suggested by working group members if not present during deliberation)*	**Consensus**	**Reasonings for exclusion (***If no reasoning provided, the measure did not meet the prescribed minimum criteria threshold (e.g. did not meet feasibility assessment, psychometric evaluation, barriers to availability or accessibility)*
A	SB x FQ	Severity of Alcohol Dependence Scale (SADS) [la escala de la intensidad de la dependencia al alcohol (EIDA)]	Excluded	-
A, S, D	SB x FQ	Severity of Dependence Scale	Excluded	5 items from the 38-item self-report burden of the set were identified. These 5-items were not recommended in consensus to add value to the overall Set (52% consensus), in addition to the already included PROMIS.
D	SB x FQ	Severity of Opiate Dependence Questionnaire (SODQ)	Excluded	Brought back in from language exclusion but was not discussed; Does not have information on 3+ reliability/validity domains, therefore propose exclude.
-		Sheehan Disability Scale (Sds)	Excluded	
A	SB x FQ	Short Alcohol Dependence Data Questionnaire (Sadd)	Excluded	
-	GF	Short Form Health Survey (SF)	Excluded	The TOP and SURE were recommended by the working group as preferred generic functioning measures.
-	GF	Short Index of Problems (SIP)	Excluded	Difficult to assess psychometrics due to large number of versions - This measure specifically attributes problems to drinking or drug use. Given that the Substance Use Recovery Evaluator (SURE) has reached consensus for inclusion in the functioning domain (with greater support than the SIP-AD) there is too much redunancy between the two to include the SIP-AD as well.
-	QoL	Smoking Cessation Quality of Life (SCQoL) Questionnaire	Excluded	-
-	-	Sociotropy-Autonomy Scale (Sas)	Excluded	-
Gambling	-	South Oaks Gambling Screen (Sogs)	Excluded	None of the identified gambling measures had definitive evidence that they were well suited as outcome measures. After consideration of psychometrics the PGSI was recommended.
Overall	MH x WB x GF	Strengths and Difficulties Questionnaire (SDQ)	Excluded	Excluded at feasibility assessment; focus on younger ages.
-	-	Structured Clinical Interview For DSM Diagnosis (SCID)	Excluded	-
-	-	Subjective Units of Distress Scale (Suds)	Excluded	-
-	-	Temperament and Character Inventory (TCI)	Excluded	-
-	-	Temporal Experience of Pleasure Scale (Teps)	Excluded	-
-	QoL x Func	The Health-Related Quality of Life for Drug Abusers Test (HRQOLDA_T)	Excluded	-
-	-	Tidslinjeskema (Danish)	Excluded	-

Areas	Domain/s	**Measure** *(Measure were suggested by working group members if not present during deliberation)*	Consensus	**Reasonings for exclusion (***If no reasoning provided, the measure did not meet the prescribed minimum criteria threshold (e.g. did not meet feasibility assessment, psychometric evaluation, barriers to availability or accessibility)*
A, D, S	SB x FQ	Timeline Follow-Back Questionnaire (TFBQ)	Excluded	Gold standard in research, but training requirement and time to administer makes this a difficult tool for practice; Reducing the relevant period to 7 days only may not be very useful.
S	SB x FQ	Tobacco Dependence Screener	Excluded	-
A, D, S	SB x FQ	Tobacco, Alcohol, Prescription Medications, and Substance Use/Misuse (TAPS) Tool	Excluded	-
-	-	Tolerance, Worried, Eye-opener, Amnesia, K/Cutdown (Modified TWEAK)	Excluded	Poor internal consistency (generally ≤ .7)
-	-	Toronto Alexithymia Scale (Tas20)	Excluded	Excluded at feasibility stage.
Gambling	SB x FQ	Victoria Gambling Screen (Vgs)	Excluded	None of the identified gambling measures had definitive evidence that they were well suited as outcome measures. After consideration of psychometrics the PGSI was recommended.
S	SB x FQ	Wisconsin Inventory of Smoking Dependence Motives (Wisdm)	Excluded	-
Overall	GF	World Health Organisation Quality of Life (Whoqol)	Excluded	-
Overall	GF	The Work and Social Adjustment Scale (WSAS)	Excluded	Discarded for breadth of coverage
Gaming	SB x FQ	Internet Gaming Disorder Test (IGDT-10)	**Included**	Selected as preferable due to its psychometric properties, as well as that it is based on the newest conceptualisations of gaming disorder, as in DSM-5. 12-month recall was recommended (77%)
Overall	QoL x GF - Generic	World Health Organisation Disability Assessment Schedule (WHODAS-12);	**Included**	Nb. WHODAS-36 Discarded for length; WHODAS-12 included;
Overall	QoL x GF - Generic	KIDSCREEN-10 [for services that exclusively treat adolescents]	**Included**	-
A, D, S	QoL x GF - Disorder specific	Substance Use Recovery Evaluator (SURE) [Also administer if problem relates to alcohol or drugs]	**Included**	Condition-specific functioning
Overall	MH x WB	Single item from PROMIS Global Health: “In general, how would you rate your physical health?”	**Included**	-
Overall	MH x WB	Two items from PROMIS Global Health: “In general, how would you rate your mental health, including your mood and your ability to think?” “How often have you been bothered by emotional problems such as feeling anxious, depressed or irritable?”	**Included**	-

Areas	Domain/s	**Measure** *(Measure were suggested by working group members if not present during deliberation)*	Consensus	**Reasonings for exclusion (***If no reasoning provided, the measure did not meet the prescribed minimum criteria threshold (e.g. did not meet feasibility assessment, psychometric evaluation, barriers to availability or accessibility)*
-	QoL x GF	Q-LES-Q	Excluded	Difficult to score; Relative lack of validation
D	QoL x GF	OSTQoL	Excluded	Specific to drug use only
D	QoL x GF	DUQOL	Excluded	Specific to drug use only

Area: A: Alcohol; D: Drugs; S: Smoking; Outcome Domains: [1]SB: Symptom Burden; [2]FQ: Frequency and Quantity of Use; [3] GF: Global Functioning and/or psychosocial functioning [4]QoL: Health related quality of life, psychosocial functioning; [5]WB: Overall Wellbeing [6]MH: Mental Health; [7]PH: Physical Health.

Case-Mix Factors

Overview

Case-mix factors are variables that moderate outcome achievement but are outside of the scope of healthcare delivery. For benchmarking to be meaningful, case-mix factors may be measured and controlled for. All case-mix factors were extracted from the studies included in the above-described systematic review. To supplement this pool of case-mix factors and to generate evidence on their strengths of associations with treatment outcomes, the project team also conducted a second, case-mix specific review of systematic reviews. Included were 39 systematic reviews that examined factors that moderate treatment success or that otherwise predict substance use or severity of dependence (see subsequent sections for the search terms, PRISMA flowchart, and list of included studies). From these, we extracted the reported links between the studied case-mix factors and the outcomes, and then synthesised these across the systematic reviews to present an overall appraisal of the strength of the link to the Working Group. The Working Group members were asked to consider the following factors when voting on each potential case-mix factor: whether it is essential (high impact on outcomes), whether it is practical to collect (low burden), and whether it is internationally comparable.

All considered case-mix factors, the measure or question used to measure the factor, and the primary reasoning for exclusion are shown in Supplementary S5.

Search strategy for systematic review of systematic reviews

PsycINFO and Medline were searched on 2nd August 2019 using the below search terms:

1	("Systematic review" or "systematic literature review" or "meta-analysis" or "meta-analytic").m_titl.
2	(smoking or smoker or smokers or tobacco or nicotine or cigarette* or ("e-cigarette*" or ecigarette* or vaping*) or (alcoholic* or alcoholism) or ((injur* or abus* or harm* or problem* or pathological or compulsive or misus* or hazardous) adj2 (substance* or drug* or alcohol* or drink* or hallucinogen* or cannabi* or marijuana* or LSD or mescaline* or psilocybin* or hash* or tetrahydrocannabinol* or THC or heroin* or opioid* or opiate* or opium* or codeine* or Dolophine* or methadose* or fentanyl* or actiq* or duragesic* or sublimaze* or morphine* or duramorph* or kadian* or avinza* or hydrocodone* or vicodin* or oxycodone* or oxycontin* or percocet* or percodan* or oxymorphone* or opana* or sedative* or hypnotic* or anxiolytic* or barbiturate* or amobarbital* or amytal* or pentobarbit* or nembutal* or phenobarbit* or luminal* or secobarbital* or seconal* or tuinal* or Benzylbutylbarbiturate* or Butalbital* or butisol* or thiopent* or benzodiazepine* or clonazepam* or klonopin or diazepam* or valium* or lorazepam* or ativan* or alprazolam* or xanax* or chlordiazepoxide* or librium* or temazepam* or restoril* or triazolam* or halcion* or midazolam* or nitrazepam* or "sleeping pill*" or zopiclone* or zolpidem* or methaqualone* or quaalude* or mandrax* or cocaine* or crack* or stimulant* or psychostimulant* or amphetamine* or methamphetamine* or methcathinone* or "bath salt*" or cathinone* or inhalant* or mdma or mda or ecstasy or GHB or gamma-hydroxybutyrate* or dissociative* or ketamine* or PCP or polysubstance* or polydrug*)) or ((dependen* or withdraw* or addict*) adj3 (substance* or drug* or alcohol* or drink* or hallucinogen* or cannabi* or marijuana* or LSD or mescaline* or psilocybin* or hash* or tetrahydrocannabinol* or THC or heroin* or opioid* or opiate* or opium* or codeine* or Dolophine* or methadose* or fentanyl* or actiq* or duragesic* or sublimaze* or morphine* or duramorph* or kadian* or avinza* or hydrocodone* or vicodin* or oxycodone* or oxycontin* or percocet* or percodan* or oxymorphone* or opana* or sedative* or hypnotic* or anxiolytic* or barbiturate* or amobarbital* or amytal* or pentobarbit* or nembutal* or phenobarbit* or luminal* or secobarbital* or seconal* or tuinal* or Benzylbutylbarbiturate* or Butalbital* or butisol* or thiopent* or benzodiazepine* or clonazepam* or klonopin or diazepam* or valium* or lorazepam* or ativan* or alprazolam* or xanax* or chlordiazepoxide* or librium* or temazepam* or restoril* or triazolam* or halcion* or midazolam* or nitrazepam* or "sleeping pill*" or zopiclone* or zolpidem* or methaqualone* or quaalude* or mandrax* or cocaine* or crack* or stimulant* or psychostimulant* or amphetamine* or methamphetamine* or methcathinone* or "bath salt*" or cathinone* or inhalant* or mdma or mda or ecstasy or GHB or gamma-hydroxybutyrate* or dissociative* or ketamine* or PCP or polysubstance* or polydrug*)) or (disorder* adj1 (substance* or drug* or alcohol* or drink* or hallucinogen* or cannabi* or marijuana* or LSD or mescaline* or psilocybin* or hash* or tetrahydrocannabinol* or THC or heroin* or opioid* or opiate* or opium* or codeine* or Dolophine* or methadose* or fentanyl* or actiq* or duragesic* or sublimaze* or morphine* or duramorph* or kadian* or avinza* or hydrocodone* or vicodin* or oxycodone* or oxycontin* or percocet* or percodan* or oxymorphone* or opana* or sedative* or hypnotic* or anxiolytic* or barbiturate* or amobarbital* or amytal* or pentobarbit* or nembutal* or phenobarbit* or luminal* or secobarbital* or seconal* or tuinal* or Benzylbutylbarbiturate* or Butalbital* or butisol* or thiopent* or benzodiazepine* or clonazepam* or klonopin or diazepam* or valium* or lorazepam* or ativan* or alprazolam* or xanax* or chlordiazepoxide* or librium* or temazepam* or restoril* or triazolam* or halcion* or midazolam* or nitrazepam* or "sleeping pill*" or zopiclone* or zolpidem* or methaqualone* or quaalude* or mandrax* or

	cocaine* or crack* or stimulant* or psychostimulant* or amphetamine* or methamphetamine* or methcathinone* or "bath salt*" or cathinone* or inhalant* or mdma or mda or ecstasy or GHB or gamma-hydroxybutyrate* or dissociative* or ketamine* or PCP or polysubstance* or polydrug*))).m_titl.
3	((gambl* adj3 (dependen* or disorder* or withdraw* or abus* or addict* or harm* or problem* or pathological or compulsive or misus* or hazardous or high-risk or risky)) or ((game* or gaming*) adj3 (dependen* or disorder* or withdraw* or abus* or addict* or harm* or problem* or pathological or compulsive or misus* or hazardous or high-risk or risky)) or (addictive behavio?r* or behavio?ral addict*)).m_titl.
4	2 or 3
5	1 and 4
6	((predict* or moderat* or determin*) adj4 outcome*).ti,ab.
7	(respon* adj4 (treatment* or outcome*)).ti,ab.
8	(relationship* or response* or respond* or predict* or moderat* or associat* or outcome* or trajector*).m_titl.
9	6 or 7 or 8
10	5 and 9

PRISMA flowchart for review of systematic reviews

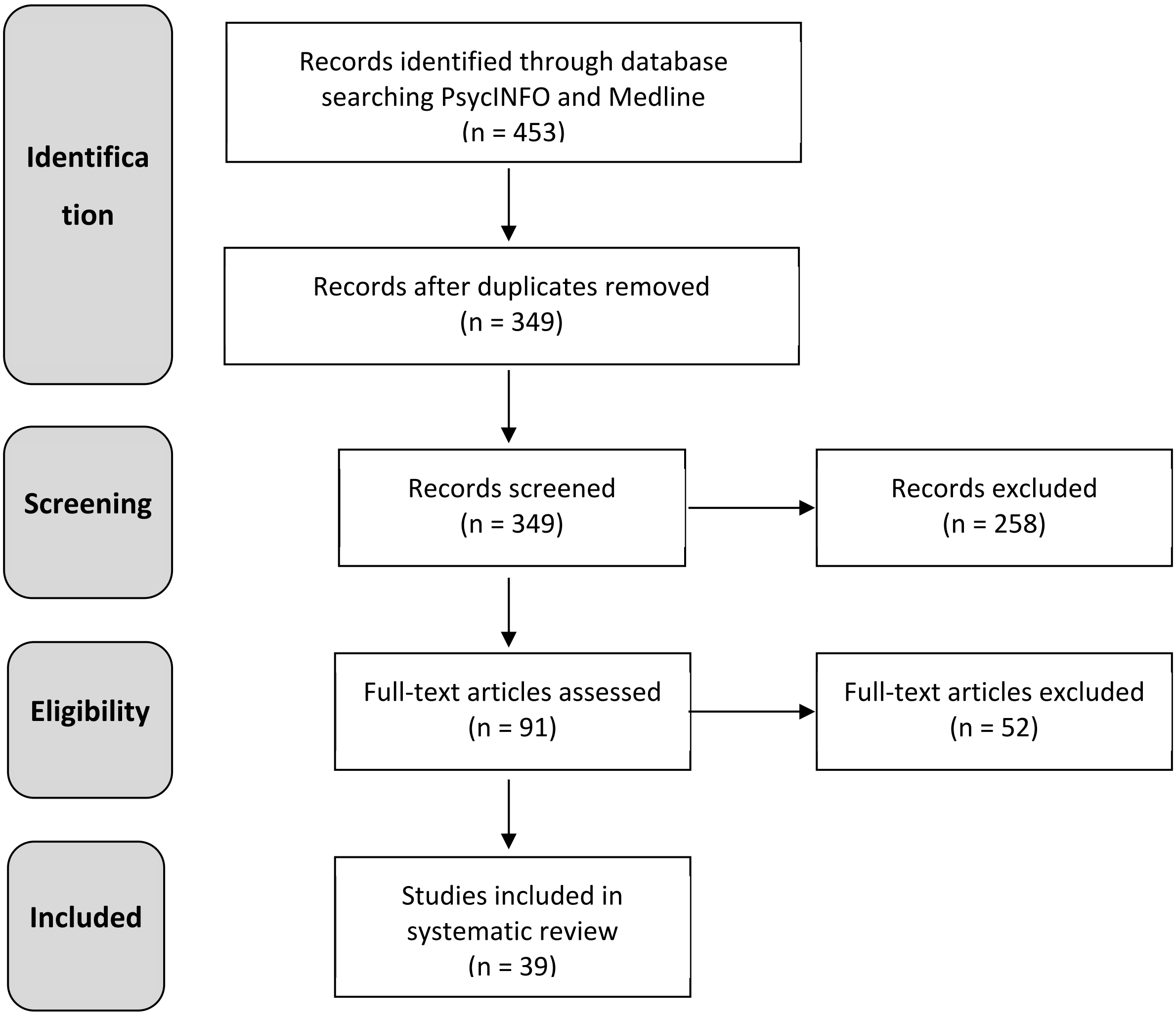

List of systematic reviews included in review of systematic reviews

1. Amlung, M. (2017). Steep delay discounting and addictive behavior: A meta-analysis of continuous associations.
2. Argyriou, E. (2017). Response inhibition and internet gaming disorder: A meta-analysis.
3. Bawor, M. (2014). Sex differences in outcomes of methadone maintenance treatment for opioid use disorder: a systematic review and meta-analysis.
4. Brewer, D. D. (1998). A meta-analysis of predictors of continued drug use during and after treatment for opiate addiction.
5. Casetta, B. (2017). Association between cigarette smoking prevalence and income level: A systematic review and meta-analysis.
6. Cengelli, S. (2012). A systematic review of longitudinal population-based studies on the predictors of smoking cessation in adolescent and young adult smokers.
7. Chaiton, M. O. (2009). A systematic review of longitudinal studies on the association between depression and smoking in adolescents.
8. Chen, D. (2015). Smoking cessation interventions for adults aged 50 or older: A systematic review and meta-analysis.
9. de Leon, J. (2005). A meta-analysis of worldwide studies demonstrates an association between schizophrenia and tobacco smoking behaviors.
10. Dennis, B. B. (2016). Pain and opioid addiction: A systematic review and evaluation of pain measurement in patients with opioid dependence on methadone maintenance treatment.
11. Durdle, H. (2008). A meta-analysis examining the relations among pathological gambling, obsessive-compulsive disorder, and obsessive-compulsive traits.
12. Fatseas, M. (2010). Relationship between anxiety disorders and opiate dependence-a systematic review of the literature: Implications for diagnosis and treatment.
13. Fluharty, M. (2017). The association of cigarette smoking with depression and anxiety: A systematic review.
14. Garbutt, J. C. (2014). Clinical and biological moderators of response to naltrexone in alcohol dependence: A systematic review of the evidence.
15. Goodson, C. M. (2014). Predictors of severe alcohol withdrawal syndrome: A systematic review and meta-analysis.
16. Goslar, M. (2019). Pharmacological treatments for disordered gambling: A meta-analysis.
17. Grabski, M. (2016). Behavioural tasks sensitive to acute abstinence and predictive of smoking cessation success: A systematic review and meta-analysis.
18. Gwaltney, C. J. (2009). Self-efficacy and smoking cessation: A meta-analysis.
19. Hitsman, B. (2003). History of depression and smoking cessation outcome: A meta-analysis.
20. Hitsman, B. (2013). Past major depression and smoking cessation outcome: A systematic review and meta-analysis update.
21. Jiang, F. (2014). Association of anxiety disorders with the risk of smoking behaviors: A meta-analysis of prospective observational studies.
22. Johnson, W. R. (2008). Beyond 'what works': A meta-analysis of substance abuse treatments for prison populations.
23. Konkoly Thege, B. (2017). Relationship between interpersonal trauma exposure and addictive behaviors: A systematic review.
24. Merkouris, S. (2016). Predictors of outcomes of psychological treatments for disordered gambling: A systematic review.
25. Ohmoto, M. (2013). Effects of moderating factors including serotonin transporter polymorphisms on smoking behavior: A systematic review and meta-analysis update.
26. Orton, S. (2018). Predictors of postpartum return to smoking: A systematic review.
27. Pallesen, S. (2007). Outcome of pharmacological treatments of pathological gambling: A review and meta-analysis.
28. Riaz, M. (2018). Predictors of smoking cessation during pregnancy: A systematic review and meta-analysis.
29. Riper, H. (2014). Effectiveness of guided and unguided low-intensity internet interventions for adult alcohol misuse: A meta-analysis.
30. Riper, H. (2018). Effectiveness and treatment moderators of internet interventions for adult problem drinking: An individual patient data meta-analysis of 19 randomised controlled trials.
31. Ruiz, M. A. (2008). Externalizing pathology and the five-factor model: A meta-analysis of personality traits associated with antisocial personality disorder, substance use disorder, and their co-occurrence.
32. Salvarli, S. I. (2019). Internet gaming disorder and its associated personality traits: A systematic review using PRISMA guidelines.
33. Solmi, M. (2016). The association between smoking prevalence and eating disorders: A systematic review and meta-analysis.
34. Turk, D. C. (2008). Predicting opioid misuse by chronic pain patients: A systematic review and literature synthesis.

35. Vangeli, E. (2011). Predictors of attempts to stop smoking and their success in adult general population samples: A systematic review.
36. Veilleux, J. C. (2019). The relationship between distress tolerance and cigarette smoking: A systematic review and synthesis.
37. Wray, J. M. (2013). A systematic review of the relationships between craving and smoking cessation.
38. Yap, M. B. (2017). Modifiable parenting factors associated with adolescent alcohol misuse: A systematic review and meta-analysis of longitudinal studies.
39. Zhang, J. (2008). Factors associated with smoking in Asian American adults: A systematic review.

Timepoints

A range of suggested timepoints was included in the Set recommendation. This was to facilitate the consistency in approaches to measurement and to emphasise, as far as possible, that collection should take place at the individual level over the full cycle of care. There is no clear consensus or available guidelines for standardising timepoints in outcomes measurement. Based on prior ICHOM Sets and discussion of core, shared timepoints across clinical pathways with the Working Group, an initial recommendation was developed. Timepoint questions are also shown in Supplementary S5.

Supplementary S5. All considered case-mix factors, the measure or question used (if included) or considered (if excluded), and primary reasoning for inclusion or exclusion.

Category	Case-Mix Factor	Measure or Question Used (if Included) or Considered (if Excluded)	Inclusion	Primary Reasoning for Exclusion
Similarly Demographic	Age	Collected using administrative data (YYYY) at baseline only.	**Included**	**-**
	Socioeconomic Status / Education	ICHOM uses a proxy of education level drawing on ISCED standards: Adults: highest level of education completed. Adolescents: proxy to be used: highest level of education completed by parents.	**Included**	**-**
	Sex at Birth	What sex were you assigned at birth?<br>o Male,<br>o Female<br>o Other________<br>o Prefer not to say	**Included**	Sex at birth did not reach consensus for inclusion last call. However, as it represents limited burden and increases comparability, this variable was included in addition to gender identity. This may also address comments raised in the last survey that in some cultures, asking about gender identity is stigmatising. In such settings, services might choose to administer only the sex at birth question, which would permit greater comparability than if neither gender nor sex were asked.
	Gender identity	What terms best express how you describe your gender identity? (Check all that apply):<br>[ ] Man [ ] Woman [ ] Non-binary [ ] Transgender<br>[ ] None of these describe me, and I,d like to specify________________<br>[ ] Prefer not to answer	**Included**	**-**
	Sexual Orientation	Do you think of yourself as: o Lesbian, Gay or Homosexual<br>o Straight or Heterosexual<br>o Bisexual<br>o Something else (Specify) ____<br>o I dont know right now<br>o Prefer not to say	**Included**	Key points raised by working group members during the calls were that: (1) There are strong associations between being of a minority sexuality and higher rates of substance use and problems, (2) There is not enough routine data collection around sexuality, and (3) If sexuality is not collected, it is difficult to know how people of minority sexualities are responding to treatment and to incorporate any sexuality-specific issues into treatment, if needed.
	Accommodation or Homelessness status	SURE: [Last week recall]"I have had stable housing" (All of the time/most of the time/a fair amount of the time/a little of the time/none of the time)	**Included**	-

	Accommodation or Homelessness status	Record accommodation status for the past four weeks:<br>1. Acute housing problem Yes [ ] No [ ]<br>At risk of eviction Yes [ ] No [ ]	**Included**	This variable is partially captured by the SURE, however there was discussion during the call over whether or not the 1 week recall period was sufficient (SURE Item): One additional suggestion is to have two brief interviewer rated items that cover the past four weeks (this is borrowed from the TOP). This would be collected at baseline.
Clinical	Trauma (Experience or History)	PTSD Screeners / Symptom measures via self-report e.g. Adverse Childhood Experiences Questionnaires or the 'Brief Trauma Questionnaire')	Excluded	1. It is not just the fact that events have occurred, but the impact of those events is also important. 2.Without therapeutic relationship/rapport it is difficult to ask about trauma (especially considering this would be collected at baseline i.e. when someone first enters a service). 3.Sensitivity of measures; short screeners likely to be highly endorsed by respondents (i.e. most people will say yes to experiencing at least 1 event and therefore the score will be meaningless).
	Mental Health - Anxiety	Brief-Assessment - GAD-2: Over the last 2 weeks, how often have you been bothered by the following problems?<br>(1) Feeling nervous, anxious or on edge (Not at all/Several days/More than half the days/Nearly every day)<br>(2) Not being able to stop or control worrying (Not at all/Several days/More than half the days/Nearly every day)	**Included**	**-**
	Mental Health - Depression	Brief-Assessment - PHQ-2: Over the last 2 weeks, how often have you been bothered by the following problems?<br>(1) Little interest or pleasure in doing things (Not at all/Several days/More than half the days/Nearly every day)<br>(2) Feeling down, depressed or hopeless (Not at all/Several days/More than half the days/Nearly every day)	**Included**	**-**
	ADHD and Conduct Disorder	Decision to include a measure and/or question for ADHD and/or Conduct Disorder.	Excluded	PROMIS Global Health is already included and has items on: General physical health, pain, fatigue, mobility, ability to do chores; General mental health (mood and ability to think), Emotional problems (Anxious, depressed, irritable); These produce two summary scores (Physical Health and Mental Health). No additional questions included.

	Age of onset/duration of use			
	Genetics and Environment	Do any of the following people you know have a problem with alcohol, drugs (including tobacco), gambling or gaming?<br>Yes; Yes, most; Yes, some; No; N/A: [My partner | My parents | People I live with |My friends]	**Included**	-
	Genetics	Have either of your biological parents or siblings had an alcohol problem? (Yes/No/Don't know);<br>Have either of your biological parents or siblings used non-prescribed drugs? (Yes/No/Don't know)	**Included**	The discussion on this variable recognised that both genetic and environmental influences or exposure are important
	Environment	Do you live with anyone who has a current alcohol problem? (Yes/No) ;<br>Do you live with anyone who currently uses non-prescribed drugs? (Yes/No)	**Included**	**-**
	Self-efficacy or motivation	Decision to include a measure or question for self-efficacy or motivation.	Excluded	It was generally felt to be important, but difficult to capture - either one item expectancy scales not being detailed enough but full scales being burdensome.
Intervention and Setting	Setting	Please indicate in which setting an intervention has taken place. Please check all that apply: Residential or inpatient treatment; Non-residential or outpatient treatment; Day treatment; Digital ; Other	**Included**	Including variables relating to treatment was voted in as important. These variables are for stratification purposes.
	Intervention Goal: Detox vs. Withdrawal	Whether an intervention is detoxification or withdrawal focused.	**Excluded**	Lacked consensus recommendation (68%)
	Intervention Type or Content	Please indicate the type of intervention. Please check all that apply: Medication for substance use (including agonist treatment); Counselling or psychotherapy; Other	**Included**	**Included**
Timepoints	'Baseline' Measurement	At assessment is recommended as a 'baseline measurement' timepoint.	**Included**	Indication of a return to baseline behaviour and recalling for further treatment;
	'End' Measurement	At each or any 'intervention end' is recommended as an 'end measurement' timepoint.	**Included**	A timepoint is needed for discharge or transition.
	Regularity of Measurement.	A minimum regularity in-between 'Baseline' and 'End' is recommended; this helps to ensure at least one additional outcome measure is made (avoiding incompletion of interventions), as well as supporting integration of measures into routine use rather than simply 'pre/post' assessment.	**Included**	**Included**
	Frequency of Measurement.	3-month frequency recommended.	**Included**	**Included**

Duration	1-year follow-up is recommended for following ‘active intervention or support’.	**Included**	Relapsing nature of condition, not done in the realm of direct service delivery but part of a funded/systems approach to longer term impact; chronic disorders that requires multiple episodes of intervention.

Open Review

Overview

Once the proposal was developed, we conducted two open online surveys to solicit feedback on the proposal: one for people with lived experience with substance use/addictive behaviour disorders (USA and UK) and one for professionals in this field (any country). The surveys were anonymous and open to any member of the public (over 18 of age) within the available geographies. The surveys ran in parallel to the surveys for the psychosis and personality disorders Sets, which aided harmonisation across these Sets (particularly with respect to a harmonised measure of global functioning). At least 70% support was required for each outcome domain and measure to consider it endorsed by the open review participants. Any potential issues or feedback raised by the open review participants were discussed and considered in the final Working Group call if the issue was new, actionable, and commonly raised. Issues included specific wording (stigmatising or unclear) and length of time to complete.

Responses were received from 34 individuals with lived experience and 123 professionals. Respondents were most commonly familiar with problems related to alcohol, tobacco, and other drugs, with fewer respondents familiar with problems related to gambling or gaming. Detailed descriptive information can be found in the following two sections.

Lived experience survey

The lived experience survey was completed by 34 participants: 31 with personal experience of problems related to alcohol, tobacco, other drugs, gambling, or gaming, and 3 with experience as a family member of someone with these conditions. The following table summarises this sample:

Characteristic	Distribution
Gender	56% male
	41% female
	3% other
Age (personal lived experience participants)	13% 18-25
	6% 26-30
	23% 31-40
	42% 41-65
	16% 66-85
Age (of individual under care of respondent)	33% 12-17
	67% Over 18
Country	71% UK
	29% USA
Problems experienced related to (not mutually exclusive categories)	71% alcohol
	41% other drugs
	35% tobacco
	3% gaming
	3% unspecified

Professional experience survey

The professional experience survey was completed by 123 participants. The following table summarises this sample:

Characteristic	Distribution
Field of experience	54% health/social care
	23% research/education
	10% advocacy/charity
	8% government/policy
	1% commercial/industry
	4% other
Geographic region	43% Oceania
	21% Europe
	15% North America
	6% Asia
	3% Africa
Most prevalent countries	46% Australia
	9% Canada
	8% USA

	7% Spain<br>6% UK
Professional experience related to (not mutually exclusive categories)	81% other drugs<br>77% alcohol<br>47% tobacco<br>21% gambling<br>15% gaming

References

1. Reeve BB, Wyrwich KW, Wu AW, Velikova G, Terwee CB, Snyder CF, et al. ISOQOL recommends minimum standards for patient-reported outcome measures used in patient-centered outcomes and comparative effectiveness research. Qual Life Res. 2013;22(8):1889-905.

2. Mapi Research Trust. ePROVIDE Databases on Clinical Outcome Assessments (COAs) Lyon, France: Mapi Research Trust,; [Available from: https://eprovide.mapi-trust.org/.

3. Üstün TB, Chatterji S, Kostanjsek N, Rehm J, Kennedy C, Epping-Jordan J, et al. Developing the World Health Organization disability assessment schedule 2.0. Bulletin of the World Health Organization. 2010;88:815-23.
